# High somatic mutations in circulating tumor DNA predict response of metastatic pancreatic ductal adenocarcinoma to first-line nab-paclitaxel plus S-1: prospective study

**DOI:** 10.1186/s12967-024-04989-z

**Published:** 2024-02-20

**Authors:** Lei Huang, Yao Lv, Shasha Guan, Huan Yan, Lu Han, Zhikuan Wang, Quanli Han, Guanghai Dai, Yan Shi

**Affiliations:** 1 Medical Center on Aging of Ruijin Hospital, MCARJH, Shanghai Jiaotong University School of Medicine, 197 Ruijin Er Road, Shanghai, 200025 China; 2 Department of Oncology, Ruijin Hospital, Shanghai Jiao Tong University School of Medicine, Shanghai, 200025 China; 3 Department of Medical Oncology, Chinese PLA General Hospital, 28 Fuxing Road, Beijing, 100853 China; 4 Department of General Surgery, Shanghai Seventh People’s Hospital, Shanghai University of Traditional Chinese Medicine, 358 Datong Road, Gaoqiao Town, Shanghai, 200137 China

**Keywords:** Metastatic pancreatic ductal adenocarcinoma (mPDAC), Circulating tumor DNA (ctDNA), High mutation allelic frequency (MAF), Nab-paclitaxel plus S-1 (NPS), Efficacy prediction, Prospective longitudinal study, Precision oncology

## Abstract

**Aims:**

We previously showed that the nab-paclitaxel plus S-1 (NPS) regimen had promising effects against metastatic pancreatic ducal adenocarcinoma (mPDAC), whose efficacy however could not be precisely predicted by routine biomarkers. This prospective study aimed to investigate the values of mutations in circulating tumor DNA (ctDNA) and their dynamic changes in predicting response of mPDAC to NPS chemotherapy.

**Methods:**

Paired tumor tissue and blood samples were prospectively collected from patients with mPDAC receiving first-line NPS chemotherapy, and underwent next-generation sequencing with genomic profiling of 425 genes for ctDNA. High mutation allelic frequency (MAF) was defined as ≥ 30% and ≥ 5% in tumor tissue and blood, respectively. Kappa statistics were used to assess agreement between mutant genes in tumor and ctDNA. Associations of mutations in ctDNA and their dynamic changes with tumor response, overall survival (OS), and progression-free survival (PFS) were assessed using the Kaplan–Meier method, multivariable-adjusted Cox proportional hazards regression, and longitudinal data analysis.

**Results:**

147 blood samples and 43 paired tumor specimens from 43 patients with mPDAC were sequenced. The most common driver genes with high MAF were *KRAS* (tumor, 35%; ctDNA, 37%) and *TP53* (tumor, 37%; ctDNA, 33%). Mutation rates of KRAS and TP53 in ctDNA were significantly higher in patients with liver metastasis, with baseline CA19-9 ≥ 2000 U/mL, and/or without an early CA19-9 response. κ values for the 5 most commonly mutated genes between tumor and ctDNA ranged from 0.48 to 0.76. MAFs of the genes mostly decreased sequentially during subsequent measurements, which significantly correlated with objective response, with an increase indicating cancer progression. High mutations of KRAS and ARID1A in both tumor and ctDNA, and of TP53, CDKN2A, and SMAD4 in ctDNA but not in tumor were significantly associated with shorter survival. When predicting 6-month OS, AUCs for the 5 most commonly mutated genes in ctDNA ranged from 0.59 to 0.84, larger than for genes in tumor (0.56 to 0.71) and for clinicopathologic characteristics (0.51 to 0.68). Repeated measurements of mutations in ctDNA significantly differentiated survival and tumor response. Among the 31 patients with ≥ 2 ctDNA tests, longitudinal analysis of changes in gene MAF showed that ctDNA progression was 60 and 58 days ahead of radiologic and CA19-9 progression for 48% and 42% of the patients, respectively.

**Conclusions:**

High mutations of multiple driving genes in ctDNA and their dynamic changes could effectively predict response of mPDAC to NPS chemotherapy, with promising reliable predictive performance superior to routine clinicopathologic parameters. Inspiringly, longitudinal ctDNA tracking could predict disease progression about 2 months ahead of radiologic or CA19-9 evaluations, with the potential to precisely devise individualized therapeutic strategies for mPDAC.

**Supplementary Information:**

The online version contains supplementary material available at 10.1186/s12967-024-04989-z.

## Introduction

Pancreatic cancer, the majority being pancreatic ductal adenocarcinoma (PDAC), is the seventh leading cause of cancer-related mortality worldwide, causing approximately 500,000 deaths in 2020 [1]. PDAC has a climbing incidence, and is highly aggressive and intractable with limited effective treatment options. Prognosis of PDAC remains grim despite therapeutic advances [2]. PDAC often has rapid progression, and is often diagnosed at an advanced stage [3]. We previously reported that metastatic PDAC (mPDAC) constituted 55%-73% of all PDAC cases at diagnosis [4–7], and had a 3-year survival rate of < 5% [8].

For metastatic PDAC (mPDAC), chemotherapy is the mainstay of management. mPDAC is characterized by poor chemo-sensitivity, with survival largely varying in patients with mPDAC receiving chemotherapy. Nano-albumin-bound (nab)-paclitaxel is an innovative drug that depletes tumor stroma through interaction between albumin and secreted acidic protein rich in cysteine. Nab-paclitaxel plus gemcitabine has been approved as the standard first-line chemotherapy for mPDAC based on the MPACT trial [9]. In our previous phase II NPSPAC trial [10], nab-paclitaxel plus S-1 (NPS) in the first-line setting showed encouraging activity and efficacy with promising objective response and survival for advanced PDAC under meticulous toxicological surveillance. We further showed that S-1 maintenance after non-progressive disease induced by first-line NPS therapy was effective and well-tolerated for patients with advanced PDAC, with encouraging survival [11].

Notably, chemotherapy is sometimes futile for mPDAC, and no markers can effectively predict response of PDAC to nab-paclitaxel-based chemotherapy. PDAC has high genetic heterogeneity across patients [12]. It is featured by diverse abundant somatic mutations, with *KRAS*, *TP53*, *SMAD4*, and *CDKN2A* being the most commonly mutated driver genes [13, 14]. More than 90% of PDACs harbor active *KRAS* mutations. Precise genetic profiling may advance individualized management of patients with mPDAC.

For mPDAC, there often exists difficulty in obtaining tumor tissue via fine-needle biopsy. Liquid biopsy, especially detection and dynamic tracking of mutations within circulating tumor DNA (ctDNA), emerges as a promising minimally-/non-invasive tumor-specific method for monitoring of treatment response and resistance, and offers advantages over conventional tissue biopsy, including cost-effectiveness, timeliness, feasibility, convenience, and repeatability [15, 16]. ctDNA is the DNA fragment released into circulation from tumor cells, and can help clinicians understand the real-time molecular events underlying cancer progression, enabling the formulation of more precise, stratified, and individualized treatment decisions and follow-up schedules to optimize patient outcomes through multidisciplinary biology-based approaches. Next Generation Sequencing (NGS) can generate valuable high-throughput genetic information when evaluating ctDNA. [17]

Previously, we showed that ctDNA had the potential to predict survival for patients with metastatic pancreatic adenocarcinoma, and that longitudinal ctDNA tracking could possibly screen cancer progression [18]. However, the conventional comparison of mutation versus non-mutation appeared to be connected with rather limited prognostic significance [18]. In this prospective study specifically focusing on patients with mPDAC receiving NPS treatment and adopting novel sensitive thresholds, we first analyzed the frequencies of mutations with high abundance in both tumor and blood samples at baseline and subsequent follow-ups and the concordance between mutations in different samples. We then explored the prognostic significance of high mutation abundance at various time points, using both univariable and multivariable methods, and revealed the predictive performances of high mutations in ctDNA. We further longitudinally analyzed the prognostic significance of the dynamic alterations of mutations in ctDNA, highlighting the lead time of ctDNA progression over radiologic progression. This study could assist with precisely predicting response of mPDAC to NPS therapy, thus aiding individualized management of mPDAC.

## Methods

### Cases

A cohort of consecutive patients with microscopically (histologically or cytologically) verified mPDAC, who were treated with first-line NPS at Chinese PLA General Hospital in Beijing, China between February 2019 and April 2020, were prospectively enrolled.

Eligibility criteria for enrollment included: (1) ≥ 18 years of age, (2) with an Eastern Cooperative Oncology Group (ECOG) Performance Status score of 0–1, (3) with life expectancy of ≥ 3 months, (4) with adequate bone marrow, renal, and liver function, (5) with paired baseline tumor (through biopsy) and blood samples available, (6) with completion of ≥ 2 cycles of NPS chemotherapy, (7) with measurable primary lesion and ≥ 1 radiologically measurable distant metastatic lesion based on the RECIST criteria (version 1.1) [19], and (8) with evaluable anticancer efficacy.

The exclusion criteria were: (1) without post-treatment imaging assessment, (2) with other cancer concurrently diagnosed, (3) NPS administered in the neoadjuvant setting, (4) receipt of radiotherapy or ablation before first-line NPS, and (5) disagreement with genetic testing.

This study was approved by the Institutional Review Board (IRB) of the Chinese PLA General Hospital. All cases provided their written informed consent to participate, receiving NPS chemotherapy as first-line treatment and providing their medical records for research analysis.

### Management

Patients with mPDAC were primarily treated with first-line NPS for ≥ 2 cycles. Nab-paclitaxel was administrated at 240 mg/m^2^ body surface area every 3 weeks and S-1 was given at 80–120 mg/m^2^ body surface area per day on days 1–14 of each 21-day cycle [11]. For patients without progression or with treatment discontinuation within 4 months during NPS treatment, S-1 monotherapy with dosing schedule as above was allowed to be administrated as maintenance therapy at the physicians’ discretion based on patients’ ECOG performance status, preference, and related efficacy and benefits. Patients were followed-up until radiologic disease progression or death of any cause. Upon cancer progression, second-line therapy, clinical trial, or best supportive care was recommended taking both patients’ performance status and tumor burden into account.

### Key variable evaluations

We recorded patient (sex, age, and ECOG score) and tumor characteristics (differentiation, location, liver, lung, bone and distant lymph node metastases, number of metastases, and conventional tumor biomarkers including CA19-9, CEA, and CA125 levels and their best changes), treatment (presence of S-1 maintenance and cycles of NPS and S-1 therapies), and outcomes (objective response and survival [OS and PFS]).

Tumor biomarkers were assessed every chemotherapy cycle. Patients were evaluated by CT or MRI scan every 2 chemotherapy cycles. Objective response was evaluated according to the RECIST criteria (version 1.1), and non-progressive disease needed to be confirmed after ≥ 4 weeks. Best objective response was assessed within 4 months after diagnosis. CA19-9 response was defined as > 50% decrease from baseline CA19-9 level within 4 cycles of NPS chemotherapy [11]. CA19-9 progression was defined as an increase in CA19-9 level compared to last measurement. For clinical assessment of a combined gene panel, ctDNA progression was defined as an increase in any of the 5 most commonly mutated genes in ctDNA (*KRAS*, *TP53*, *CDKN2A*, *SMAD4*, and *ARID1A*) compared to last measurement or new emergence of any of these 5 genes in ctDNA.

### Samples collection

As previously described [18], we collected malignant tissues through biopsy, and peripheral blood samples at baseline before the first cycle of chemotherapy, subsequently right before each new chemotherapy cycle, and right after the last cycle for mutations profiling. We prepared plasma samples for DNA extraction ≤ 2 h after blood collection. Then, we collected the white blood cells (WBCs) from the buffy coat of the identical individual for sequencing as normal controls to screen mutations due to clonal hematopoiesis and germline mutations. The mean coverage depth of WBCs sequencing was ~ 300 ×.

### DNA extraction and targeted next-generation sequencing (NGS)

As detailed previously [18], we collected tissue samples after Proteinase K digestion and blood samples into EDTA tubes. We extracted fresh tumor samples using DNeasy Blood & Tissue Kit (Qiagen, Germany), and separated plasma using centrifugation at 3000×*g* for 10 min. Genomic DNA was extracted from digested tissue and plasma using the QIAmp Circulating Nucleic Acid Kit (Qiagen), and also from WBCs using the DNeasy Blood & Tissue Kit as normal control. We determined the A260/A230 and A260/A280 ratios of purified genomic DNA using Nanodrop2000 (Thermo Fisher Scientific), and quantified all DNA samples by Qubit 3.0 using the dsDNA HS Assay Kit (Life Technologies).

We used the KAPA Hyper Prep kit (KAPA Biosystems) to prepare sequencing libraries, and used Covaris M220 (Covaris) to shear genomic DNA strands into 350-bp fragments, which were A-tailored and then sequentially ligated with indexed sequencing adapters. To maximize retrieval of ctDNA from plasmas, up to 50 ng of ctDNA were purified using the Agencourt AMPure XP beads (Beckman Coulter; for size sorting), sequentially followed by end-repairing, A-tailing, ligation with customized adapters which contained unique molecular indices, and PCR amplification with primers which contained de-multiplexing indices. We used the Agencourt AMPure XP beads to purify the PCR-amplified libraries, and pooled together up to 2 g of total library input from different libraries with unique indexes.

We added XGen Universal blocking oligos (Integrated DNA Technologies) and human cot-1 DNA (Life Technologies) as blocking reagents, and used Dynabeads M-270 (Life Technologies) and XGen Lockdown Hybridization and Wash Kit (Integrated DNA Technologies) for the capture reaction. Captured libraries were on-beads PCR-amplified using Illumina p5 (5′ to 3′, AATGATACGGCGACCGA) and p7 primers (5′ to 3′, CAAGCAGAAGACGGCATACGAGAT) in KAPA HiFi HotStart ReadyMix (KAPA Biosystems), followed by purification using the Agencourt AMPure XP beads.

Libraries were quantified using the KAPA Library Quantification kit (KAPA Biosystems), and the library fragment size was determined using Bioanalyzer 2100 (Agilent Technologies). The target-enriched library was sequenced on the HiSeq4000 NGS platform (Illumina) [20, 21], and the NGS panel included 425 genes. [18]

### Data processing and bioinformatics

Processing of the sequencing data followed previously established procedures [20, 22, 23]. Sequencing reads in the FASTQ format were generated by base calling using BCL2FASTQ (Illumina, Inc.). We used the open-source software TRIMMOMATIC48 [24] for quality control (QC) and removal of terminal adapter sequences, and removed leading/trailing low-quality data (quality reading score < 30) or N bases from the FASTQ files. We used the Burrows-Wheeler Aligner (BWA-mem, version 0.7.17) alignment algorithm [25] to align sequencing data and map the filtered sequencing reads to the reference human genome sequence GRCh37 with default parameters. We used Picard to mark and remove duplicate reads, followed by realignment of de-duplication reads at intervals with mismatches around known insertions/deletions (Indels) and recalibration of base quality scores using Genome Analysis Toolkit (GATK) 3.4.0. We excluded samples with contamination rates > 0.02 or Total Q Scores < 35. We detected single nucleotide variants (SNVs), somatic mutations and Indels with tumor and matched normal DNA using VARSCAN2 (version 2.3.9) [26], with the minimum variant allele frequency (VAF) threshold set at 0.1%. Variants with frequency > 1% of the population in the dbSNPs and 1000 Genomes Project [27], the Genome Aggregation Database [28], and the Exome Aggregation Consortium [29] were excluded. SNVs/indels were annotated using ANNOVAR and manually checked with the Integrative Genomics Viewer (IGV).

To sensitively and specifically identify mutations of low abundance in ctDNA and to eliminate sequencing artifacts, we applied further filtering criteria to variants and a customized library preparation with a bi-barcoding system named Automated Triple Groom Sequencing (ATG-Seq) [30] was utilized to process ctDNA samples. To assemble a position- and base substitution-specific background error database based on allele frequency and distinct supporting reads throughout the panel, a bioinformatics polishing pipeline was constructed via sequencing a pool of plasma samples collected from 40 healthy donors. We considered an alternation as sequencing noise if its allele frequency and distinct supporting reads were not significantly higher than the corresponding background errors in the database. To minimize the errors from PCR, hybridization, damaging, sequencing, and contamination, and to avoid mutations from nontumor sources in ctDNA, the following procedures were performed: (1) the ctDNA fragment was sequenced at a depth of ~ 5000, which produced redundant DNA molecules; (2) we used mapping positions and a bi-barcode system to maximize the representative power of unique DNA molecules; and (3) we used a duplex-assisted decoder system to filter mapping and sequencing artifacts. We also analyzed genomic DNA from the WBCs of the buffy coat after plasma separation as the normal control sample for germline and clonal hematopoiesis mutation filtering. We defined ctDNA positivity as follows: (1) supporting reads ≥ 3 and total reads ≥ 100 for the mutations detected in tissues; and (2) supporting reads ≥ 6 and total reads ≥ 100 for the mutations undetected in tissues.

High mutation in tissue was defined as mutation allelic frequency (MAF) ≥ 30.0%, high mutation in ctDNA as MAF ≥ 5.0%, and high mutation abundance change as MAF change ≤ − 2.0% [31]. High and low mutations of the 7 most commonly mutated genes in tumor and blood tissues for each individual were illustrated using heat plot.

### Statistics

Categorical data were shown as count (percentage [%]), and compared between the high and low/no ctDNA mutation groups using *χ*^2^ or Fisher’s exact test where appropriate, with the corresponding histograms illustrated. Continuous variables were shown as median (interquartile range), and compared between long and short survival groups using Wilcoxon test, with the corresponding violin plots illustrated.

Correlations between mutations in tumor and ctDNA were analyzed using Pearson correlation, with the correlation coefficient *r* calculated. Concordances of high, low, and no mutations between tumor and blood samples were further assessed using confusion matrix analysis, with the *κ* statistic computed. Dynamic alterations of mutations in ctDNA were illustrated using line charts.

Overall survival (OS) was defined as the time interval between diagnosis and death from any cause or last follow-up, and progression-free survival (PFS) was calculated from diagnosis through local or distant disease progression, death, or last follow-up, whichever occurred first. Survivals of individuals with high versus low/no mutations were first depicted using waterfall plots. Univariable survival was computed using the Kaplan–Meier method and compared between ctDNA mutations using the log-rank test.

Associations of mutations in tumor and ctDNA with OS and PFS were then explored using multivariable-adjusted Cox proportional hazards regression, with hazard ratios (HRs) and corresponding 95% confidence intervals (CIs) computed. The adjusted factors included sex, age, ECOG performance status, tumor differentiation grade, primary tumor location, liver metastasis, lung metastasis, bone metastasis, distant lymph node metastasis, CA19-9 level, CEA level, CA125 level, cycle of chemotherapy, and S-1 maintenance therapy. We verified the proportional hazards assumption both analytically using the scaled Schoenfeld residuals test and graphically using the log–log plot before survival modelling [32]. Bootstraps of the Cox proportional regression analyses (number of bootstrap replicates = 200) were performed for internal validation.

Associations of repeatedly measured mutations in ctDNA with OS, PFS, and best objective response were evaluated using longitudinal data analysis [33]. We further quantified and compared the performances of mutations in tumor and ctDNA and routine patient and tumor characteristics for predicting 6-month OS and PFS using receiver operating characteristic (ROC) curve analysis, with the area under the curve (AUC) computed. To evaluate the clinical net benefits of studied genes, decision curve analysis (DCA) according to Vickers et al. [34] was performed. We performed statistical analyses using R software (version 4.2.3), with statistical significance defined as 2-sided *P* < 0.05.

## Results

### Baseline characteristics

From February 1, 2019 through April 30, 2020, a total of 159 patients with mPDAC were treated in the Chinese PLA General Hospital, and 78 patients were managed with first-line NPS chemotherapy, with evaluable antitumor efficacy. For eligible patients with both tumor tissues and blood samples available at baseline (n = 43), NGS was performed to detect cancer-related gene mutations. 31 cases had peripheral blood samples subsequently collected, and a total of 147 blood samples underwent NGS.

In the total cohort (n = 43; Table 1), male proportion was 74%, and the median age was 57 years. 21% of the patients had an ECOG performance status score of 1. Most of the cancers were moderately-poorly or poorly differentiated (60%) and located at pancreas tail (51%). Proportions of cases with liver, lung, bone, and distant lymph node metastasis were 93%, 19%, 28%, and 44%, respectively. 70% of the patients had ≥ 2 metastases. 91% of the patients had an elevated CA19-9 level, and 58% had a CA19-9 level of ≥ 2000 U/mL. During follow-up, 81% of the patients had a decreased CA19-9 level, and 42% had a decline > 50% compared to baseline. CEA and CA125 levels were abnormal in 65% and 70% of the patients at baseline, and decreased in 72% and 79% subsequently. The median cycle of NPS chemotherapy was 4. Partial response was achieved in 44% of the patients as best objective response. 30% of the cases received S-1 maintenance (median cycle = 2). The median OS was 10 months, and 79% and 49% of the patients had an OS ≥ 6 and ≥ 12 months, respectively; the median PFS was 6 months, and 49% and 23% of the cases had a PFS ≥ 6 and ≥ 12 months, respectively. Characteristics of the repeatedly measured cohort (n = 31) were similar to the total cohort (Table 1).

**Table 1:** Baseline characteristics

Variable	Category	Total	Repeated measured
n		43	31
Sex	Male	32 (74.4)	22 (71.0)
Age, years		57 (50–63)	57 (50–62)
ECOG PS score	1	9 (20.9)	6 (19.4)
Tumor differentiation	Good	1 (2.3)	0 (0.0)
	Moderate	13 (30.2)	11 (35.5)
	Moderate-poor	17 (39.5)	13 (41.9)
	Poor	12 (27.9)	7 (22.6)
Tumor location	Pancreas head	11 (25.6)	11 (35.5)
	Pancreas body	8 (18.6)	3 (9.7)
	Pancreas tail	22 (51.2)	15 (48.4)
	Pancreas body and tail	2 (4.7)	2 (6.5)
Liver metastasis	Yes	40 (93.0)	30 (96.8)
Lung metastasis	Yes	8 (18.6)	4 (12.9)
Bone metastasis	Yes	12 (27.9)	10 (32.3)
Distant lymph node metastasis	Yes	19 (44.2)	13 (41.9)
Number of metastases	≥ 2	30 (70.0)	20 (64.5)
Baseline CA19-9 level, U/mL		4527.0 (932.2–16,580.0)	3488.5 (900.1–11,563.5)
	Abnormal	39 (90.7)	28 (90.3)
	≥ 2000	25 (58.1)	17 (54.8)
Best CA19-9 change, U/mL		− 60.8 (− 94.7 to − 16.7)	− 60.8 (− 94.3 to − 16.7)
	< 0	35 (81.4)	25 (80.7)
	> 50% decline from baseline	18 (41.9)	14 (45.2)
Baseline CEA level, µg/L		21.0 (10.1–41.0)	22.6 (10.9–42.1)
	Abnormal	28 (65.1)	20 (64.5)
Best CEA change, µg/L		− 17.5 (− 63.6 to 143.4)	24.3 (− 62.6 to 168.2)
	< 0	31 (72.1)	20 (64.5)
Baseline CA125 level, U/mL		138.0 (72.5–488.2)	136.2 (77.5–346.3)
	Abnormal	30 (69.8)	22 (71.0)
Best CA125 change, U/mL		− 33.8 (− 68.2 to 45.8)	− 17.4 (− 66.1 to 53.8)
	< 0	34 (79.1)	23 (74.2)
Cycle of chemotherapy		4 (3–6)	4 (3–6)
Best objective response	Partial response	19 (44.2)	14 (45.2)
	Stable disease	14 (32.6)	11 (35.5)
	Progressive disease	10 (23.3)	6 (19.4)
S-1 maintanence	Yes	13 (30.2)	8 (25.8)
Cycle of S-1		2 (2–9)	3 (2–7)
Overall survival, months	Median	9.8 (8.6–NE)	10.0 (9.1–NE)
	≥ 6	34 (79.1)	25 (80.7)
	≥ 12	21 (48.8)	16 (51.6)
Progression-free survival, months	Median	5.9 (4.4–7.4)	6.1 (4.8–8.6)
	≥ 6	21 (48.8)	17 (54.8)
	≥ 12	10 (23.3)	8 (25.8)

Continuous variables are shown as median (interquartile range), and categorical variables as count (percentage [%])

*ECOG PS* Eastern Cooperative Oncology Group Performance Status, *CI* confidence interval, *NE* not estimable

### Mutations in tumor and blood (ctDNA) samples at baseline

**Fig. 1:**
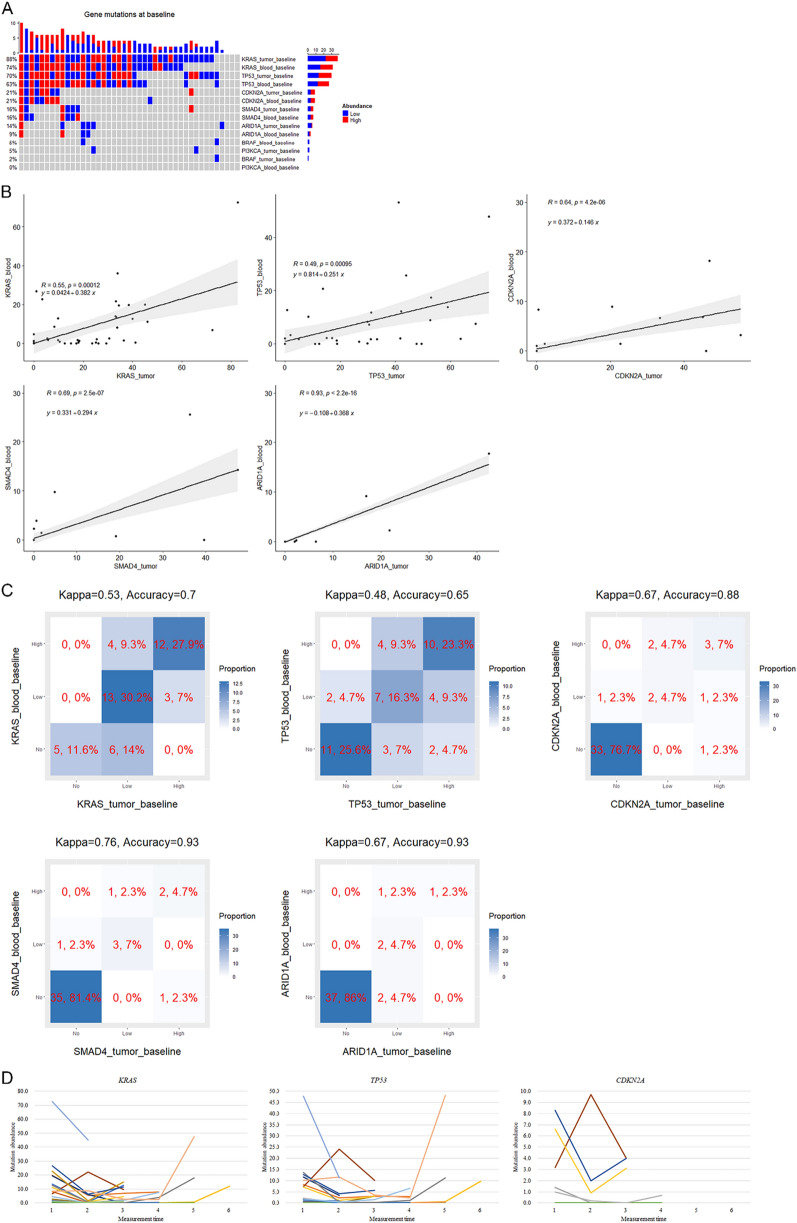
Mutations at baseline and temporal changes. (**A**) Mutation statuses (high, low, and no) of the most commonly mutated genes in tumor and ctDNA. (**B**) Pearson correlations between mutations in tumor and ctDNA. (**C**) Concordance between high, low, and no mutations in tumor and ctDNA. (**D**) Dynamic changes of mutation abundances in ctDNA

Evaluable NGS findings suggesting pathogenic somatic mutations in ctDNA were obtained in all of the baseline tumor and blood samples. For each analyzed case, the genes with the largest mutation abundance and the other somatic mutated genes are shown in Additional file 1: Table S1. In the total cohort (Table 2 and Fig. 1A), the median tumor mutation burden (TMB) was 5 mutations per million bases. *KRAS* and *TP53* mutations were the most common cancer-related mutations in both tumor and ctDNA samples. The most common *KRAS* mutation type in tumor was *G12D* (51%), followed by *G12V* (26%). *KRAS* was mutated in 88% and 74% of the tumor and blood samples, respectively, with high mutation abundance (MAF ≥ 30% and ≥ 5% in tumor and ctDNA samples, respectively) [31] observed in 35% and 37% of the samples. *TP53* was mutated in 70% and 63% of the tumor and ctDNA samples, respectively, with high mutation abundance seen in 37% and 33% of the samples. For the other key driver genes for PDAC, the mutation frequency of *CDKN2A* was 21% in both the tumor and blood samples, with high mutation abundance observed in 12% of both samples, and the mutation frequency of *SMAD4* was 16% in both the tumor and ctDNA samples, with high mutation abundance seen in 7% of both samples. For the other genes, *ARID1A* was mutated in 14% and 9% of the tumor and blood samples, respectively, with high mutation abundance observed in 2% and 5% of the samples. The mutation frequencies of *BRAF* in the tumor and blood samples were 2% and 5%, respectively, and the mutation frequencies of *PI3KCA* in the tumor and ctDNA samples were 5% and 0%, respectively. The median numbers of any mutated genes in the tumor and blood samples were 5 and 3, respectively, and among them, the number of mutated driver genes in both samples was 2. 21% of the patients had germline mutation.

**Table 2:** Mutations in blood and tumor samples at baseline

Variable	Category	Total	Repeatedly measured
*Baseline measurement*			
n		43	31
Tumor mutation burden (mutations/mb)		5.4 (3.3–6.7)	4.9 (3.3–6.7)
* KRAS* mutation type in tumor	*G12D*	22 (51.2)	16 (51.6)
	*G12V*	11 (25.6)	7 (22.6)
	Others	5 (11.6)	3 (9.7)
	Not mutated	5 (11.6)	5 (16.1)
* KRAS* mutation in tumor	Yes	38 (88.4)	26 (83.9)
* KRAS* mutation abundance in tumor (%)^a^		25.8 (12.5–34.4)	23.9 (10.5–36.4)
* KRAS* mutation abundance in tumor ≥ 30.0%	Yes	15 (34.9)	10 (32.3)
* KRAS* mutation in blood	Yes	32 (74.4)	23 (74.2)
* KRAS* mutation abundance in blood (%)^aa^		5.8 (1.5–16.7)	4.7 (1.4–13.6)
* KRAS* mutation abundance in blood ≥ 5.0%	Yes	16 (37.2)	11 (35.5)
* TP53* mutation in tumor	Yes	30 (69.8)	20 (64.5)
* TP53* mutation abundance in tumor (%)^a^		30.8 (15.0–47.7)	30.2 (16.0–51.1)
* TP53* mutation abundance in tumor ≥ 30.0%	Yes	16 (37.2)	10 (32.3)
* TP53* mutation in blood	Yes	27 (62.8)	19 (61.3)
* TP53* mutation abundance in blood (%)^a^		7.2 (1.8–12.7)	3.2 (1.9–10.2)
* TP53* mutation abundance in blood ≥ 5.0%	Yes	14 (32.6)	9 (29.0)
* CDKN2A* mutation in tumor	Yes	9 (20.9)	7 (22.6)
* CDKN2A* mutation abundance in tumor (%)^a^		33.4 (20.4–45.9)	33.4 (2.1–45.9)
* CDKN2A* mutation abundance in tumor ≥ 30.0%	Yes	5 (11.6)	4 (12.9)
* CDKN2A* mutation in blood	Yes	9 (20.9)	7 (22.6)
* CDKN2A* mutation abundance in blood (%)^a^		6.6 (1.4–8.3)	3.2 (1.4–6.8)
* CDKN2A* mutation abundance in blood ≥ 5.0%	Yes	5 (11.6)	3 (9.7)
* SMAD4* mutation in tumor	Yes	7 (16.3)	4 (12.9)
* SMAD4* mutation abundance in tumor (%)^a^		19.7 (1.7–39.6)	10.4 (1.2–29.4)
* SMAD4* mutation abundance in tumor ≥ 30.0%	Yes	3 (7.0)	1 (3.2)
* SMAD4* mutation in blood	Yes	7 (16.3)	4 (12.9)
* SMAD4* mutation abundance in blood (%)^a^		3.9 (1.5–14.3)	1.9 (1.2–3.1)
* SMAD4* mutation abundance in blood ≥ 5.0%	Yes	3 (7.0)	0 (0.0)
* ARID1A* mutation in tumor	Yes	6 (14.0)	4 (12.9)
* ARID1A* mutation abundance in tumor (%)^a^		11.7 (2.3–21.8)	4.4 (2.2–14.1)
* ARID1A* mutation abundance in tumor ≥ 30.0%		1 (2.3)	0 (0.0)
* ARID1A* mutation in blood	Yes	4 (9.3)	2 (6.5)
* ARID1A* mutation abundance in blood (%)^a^		5.7 (1.2–13.4)	1.2 (0.2–2.2)
* ARID1A* mutation abundance in blood ≥ 5.0%	Yes	2 (4.7)	0 (0.0)
* BRAF* mutation in tumor	Yes	1 (2.3)	1 (3.2)
* BRAF* mutation in blood	Yes	2 (4.7)	2 (6.5)
* PI3KCA* mutation in tumor	Yes	2 (4.7)	1 (3.2)
* PI3KCA* mutation in blood	Yes	0 (0.0)	0 (0.0)
Number of any mutated genes in tumor		5 (4–7)	5 (4–7)
Number of any mutated genes in blood		3 (2–5)	4 (2–5)
Number of mutated driver genes in tumor		2 (2–3)	2 (2–3)
Number of mutated driver genes in blood		2 (1–3)	2 (1–3)
Germline mutation	Yes	9 (20.9)	7 (22.6)

^a^Computed for the respective gene with mutation in the respective specimen

The mutation rate of *KRAS* in ctDNA was significantly higher in patients with baseline CA19-9 ≥ 2000 U/mL (92% vs 50%) and those with liver metastasis (82% vs 33%) compared with their counterparts, and the mutation rate of *TP53* in ctDNA was significantly higher in patients with liver metastasis (71% vs 0%). In patients with baseline CA19-9 ≥ 2000 U/mL (54% vs 13% and 46% vs 6%, respectively) and those without an early decline in CA19-9 level > 50% compared with baseline, namely, CA19-9 response (75% vs 21% and 67% vs 13%, respectively), the high mutation (MAF ≥ 5%) rates of *KRAS* and *TP53* in ctDNA were significantly higher than their counterparts.

The mutation allelic frequencies (MAFs) of *KRAS* (*r* = 0.55), *TP53* (*r* = 0.49), *CDKN2A* (*r* = 0.64), *SMAD4* (*r* = 0.69), and *ARID1A* (*r* = 0.93) in the tumor and blood samples were significantly correlated with each other in a linear manner (Fig. 1B). Correspondingly, after dividing the MAFs into 3 categories (no mutation, low mutation abundance, and high mutation abundance), the κ values for the 5 genes between the tumor and blood samples were 0.53, 0.48, 0.67, 0.76, and 0.67, respectively, with the accuracies being 0.70, 0.65, 0.88, 0.93, and 0.93, respectively (Fig. 1C).

### Dynamic changes of mutations in ctDNA and their associations with best objective response within 4 months after diagnosis

At the second measurement (Table 3 and Fig. 1D), the proportions of *KRAS* mutation and high mutation in ctDNA dropped to 48% and 19%, respectively. *KRAS* mutation abundance increased in only 10% of the patients, who all experienced progressive disease (PD) within 4 months after diagnosis. 36% of the patients had a decrease in *KRAS* mutation abundance ≥ 2% (high abundance decrease), among whom 82% achieved partial response (PR) as best objective response, and the others had stable disease (SD). The proportions of *TP53* mutation and high mutation decreased to 42% and 10%, respectively. *TP53* mutation abundance showed an elevation in only 7% of the cases, who both experienced PD at 1 and 3 months, respectively. 29% of the patients had a reduction in *TP53* mutation abundance ≥ 2%, among whom 89% achieved PR, and the other had SD. The proportions of *CDKN2A* mutation and high mutation dropped to 13% and 3%, respectively. *CDKN2A* mutation abundance increased in only 3% of the patients, who experienced PD at 2 months. 7% of the patients showed a decrease in *CDKN2A* mutation abundance ≥ 2%, who both achieved PR.

**Table 3:** Mutations and changes in blood samples for the 2nd and 3rd measurements

Variable	Category	Repeatedly measured
*2nd measurement*		
* KRAS* mutation in blood	Yes	15 (48.4)
* KRAS* mutation abundance in blood (%)^a^		1.1 (0.5–6.5)
* KRAS* mutation abundance in blood ≥ 5.0%	Yes	6 (19.4)
Elevated *KRAS* mutation abundance in blood^b^	Yes	3 (9.7)
Change of *KRAS* mutation abundance in blood (%)^b^		− 1.7 (− 11.9 to − 0.3)
Change of *KRAS* mutation abundance in blood^b^ ≤ − 2.0%	Yes	11 (35.5)
* TP53* mutation in blood	Yes	13 (41.9)
* TP53* mutation abundance in blood (%)^a^		1.2 (0.4–4.2)
* TP53* mutation abundance in blood ≥ 5.0%	Yes	3 (9.7)
Elevated *TP53*mutation abundance in blood^b^	Yes	2 (6.5)
Change of *TP53*mutation abundance in blood (%)^b^		− 1.9 (− 6.0 to − 0.1)
Change of *TP53*mutation abundance in blood^b^ ≤ − 2.0%	Yes	9 (29.0)
* CDKN2A* mutation in blood	Yes	4 (12.9)
* CDKN2A* mutation abundance in blood (%)^a^		1.5 (0.6–5.9)
* CDKN2A* mutation abundance in blood ≥ 5.0%	Yes	1 (3.2)
Elevated *CDKN2A* mutation abundance in blood^b^	Yes	1 (3.2)
Change of *CDKN2A* mutation abundance in blood (%)^b^		− 1.1 (− 5.7 to 0.0)
Change of *CDKN2A* mutation abundance in blood^b^ ≤ − 2.0%	Yes	2 (6.5)
*3rd measurement*		
* KRAS* mutation in blood	Yes	12 (38.7)
* KRAS* mutation abundance in blood (%)^a^		3.7 (1.9–10.6)
* KRAS* mutation abundance in blood ≥ 5.0%	Yes	5 (16.1)
Elevated *KRAS* mutation abundance in blood^b^	Yes	1 (3.2)
Change of *KRAS* mutation abundance in blood (%)^b^		− 1.7 (− 6.6 to − 0.8)
Change of *KRAS* mutation abundance in blood^b^ ≤ − 2.0%	Yes	9 (29.0)
* TP53* mutation in blood	Yes	9 (29.0)
* TP53*mutation abundance in blood (%)^a^		3.1 (1.5–3.6)
* TP53*mutation abundance in blood ≥ 5.0%	Yes	2 (6.5)
Elevated *TP53*mutation abundance in blood^b^	Yes	2 (6.5)
Change of *TP53*mutation abundance in blood (%)^b^		− 1.9 (− 4.7 to − 0.3)
Change of *TP53*mutation abundance in blood^b^ ≤ − 2.0%	Yes	8 (25.8)
* CDKN2A* mutation in blood	Yes	3 (9.7)
* CDKN2A* mutation abundance in blood (%)^a^		4.0 (3.1–4.0)
* CDKN2A* mutation abundance in blood ≥ 5.0%	Yes	0 (0.0)
Elevated *CDKN2A* mutation abundance in blood^b^	Yes	1 (3.2)
Change of *CDKN2A* mutation abundance in blood (%)^b^		− 1.2 (− 3.5 to 0.0)
Change of *CDKN2A* mutation abundance in blood^b^ ≤ − 2.0%	Yes	2 (6.5)

^a^Computed for the respective gene with mutation in the respective specimen

^b^Compared with baseline

At the third measurement (Table 3 and Fig. 1D), the proportions of *KRAS* mutation and high mutation in ctDNA further dropped to 39% and 16%, respectively. Compared to baseline, *KRAS* mutation abundance increased in only 3% of the patients, who experienced PD at 3 months. 29% of the patients had a high abundance decrease in *KRAS* mutation, among who 89% achieved PR, and the other had SD. The proportions of *TP53* mutation and high mutation further decreased to 29% and 7%, respectively. *TP53* mutation abundance showed an elevation still in 7% of the cases, who both experienced PD. 26% of the patients had a reduction in *TP53* mutation abundance ≥ 2%, among whom 88% achieved PR, and the other had SD. The proportions of *CDKN2A* mutation and high mutation dropped to 10% and 0%, respectively. *CDKN2A* mutation abundance increased in still 3% of the patients, who experienced PD. Still, 7% of the patients showed a decrease in *CDKN2A* mutation abundance ≥ 2%, who both achieved PR.

### Associations of mutations in ctDNA at baseline with survival of patients treated with NPS

**Fig. 2:**
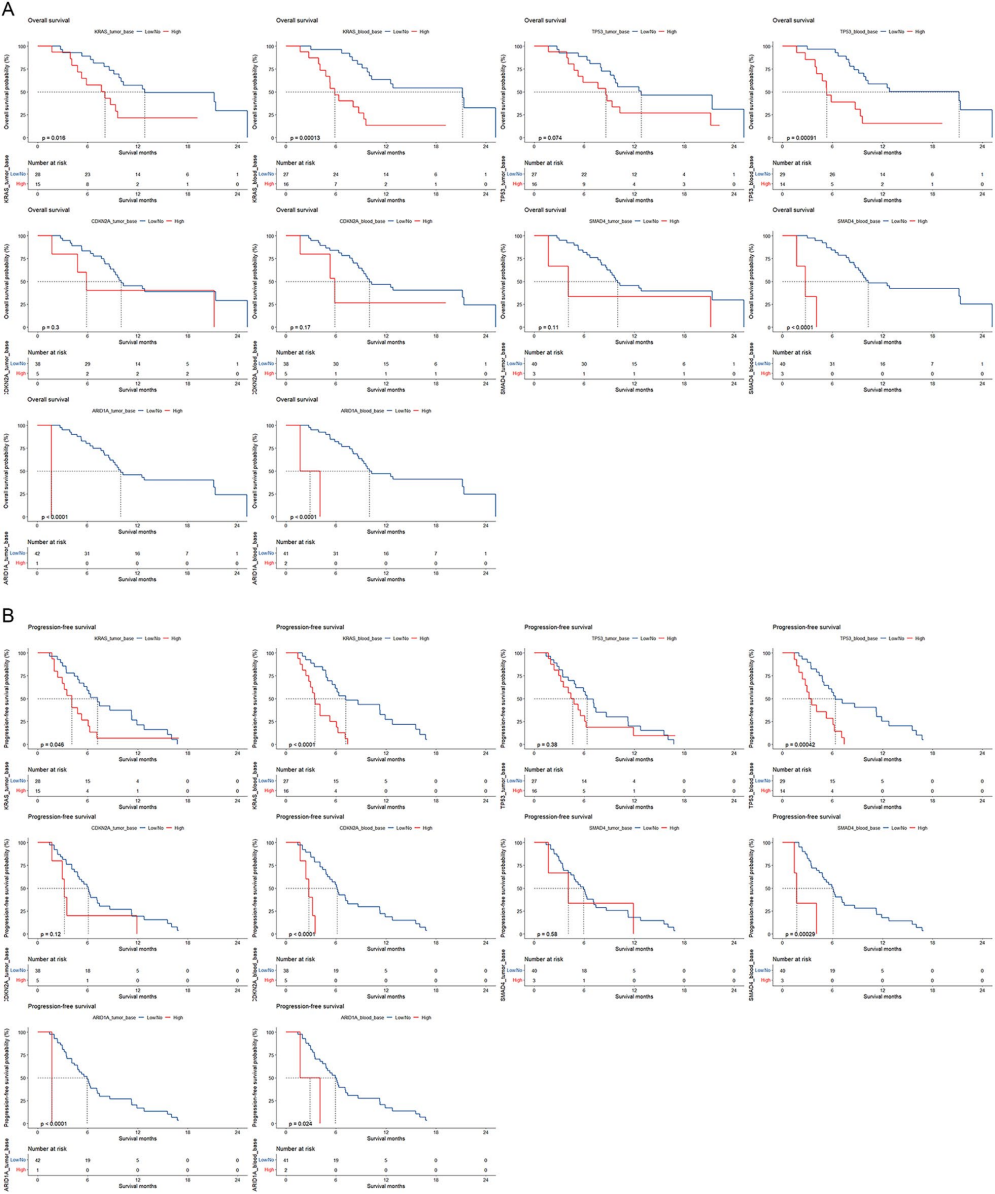
Kaplan–Meier plots for associations of mutations in tumor and ctDNA at baseline with overall survival (**A**) and progression-free survival (**B**)

In the 43 cases with available baseline tumor and blood samples (Fig. 2), high *KRAS* mutation abundances in both tumor (OS, *P* = 0.016; PFS, *P* = 0.046) and blood samples (OS, *P* < 0.001; PFS, *P* < 0.001) were significantly associated with poorer survival. Patients with high *TP53* mutation abundance in ctDNA significantly had worse survival (OS, *P* < 0.001; PFS, *P* < 0.001), while mutation abundance in tumor tissue was not significantly associated with survival. For the associations of *CDKN2A* mutations in tumor and blood samples with survival, only high *CDKN2A* mutation abundance in ctDNA was linked to inferior PFS (*P* < 0.001). While *SMAD4* mutation abundance in tumor was not significantly prognostic, high *SMAD4* mutation abundance in ctDNA was significantly associated with poorer survival (OS, *P* < 0.001; PFS, *P* < 0.001). Cases with high *ARID1A* mutation abundance in both tumor (OS, *P* < 0.001; PFS, *P* < 0.001) and blood samples (OS, *P* < 0.001; PFS, *P* = 0.024) significantly had worse survival.

**Fig. 3:**
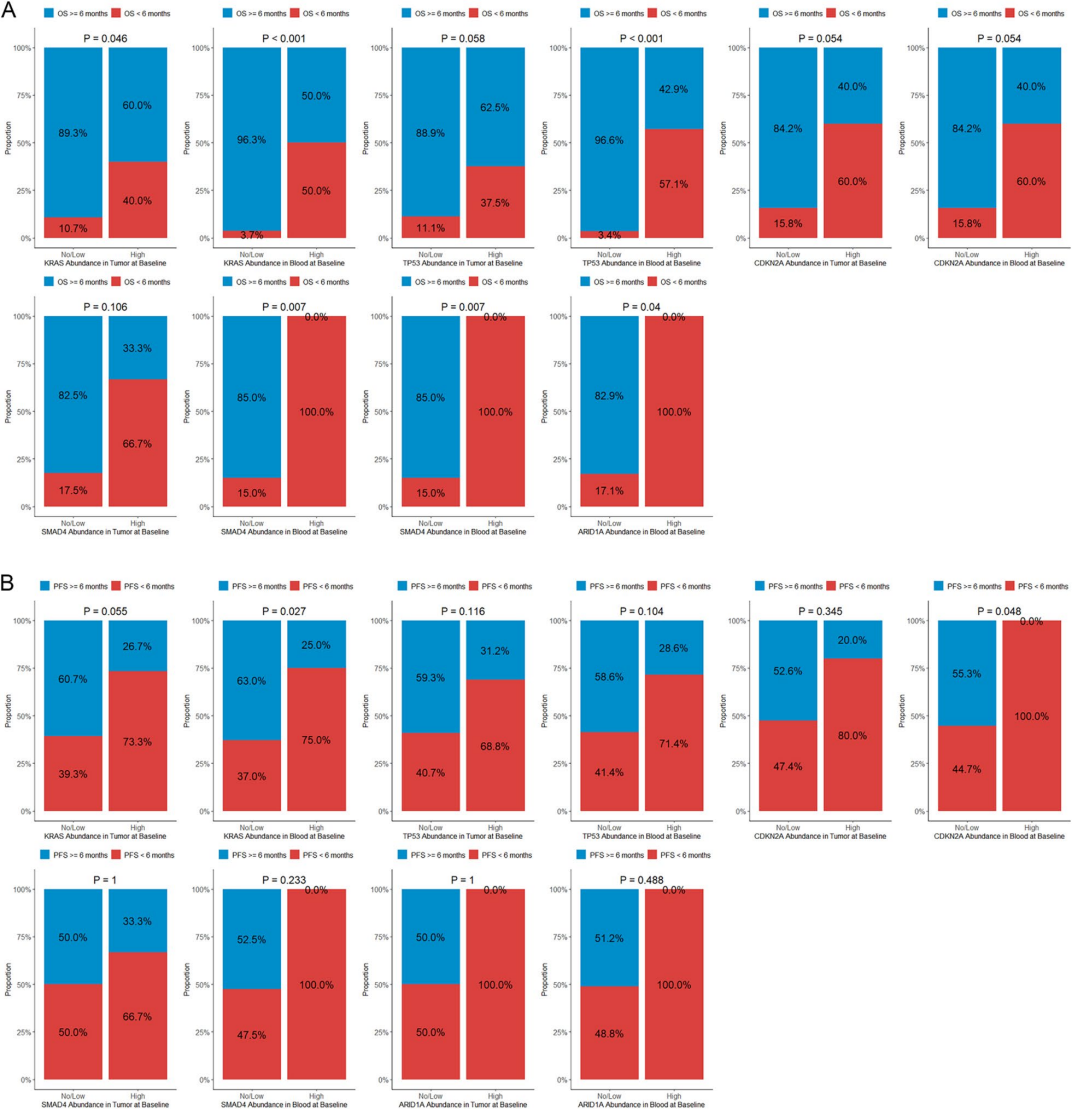
Proportions of cases with overall survival (OS; **A**) and progression-free survival (PFS; **B**) < versus ≥ 6 months in patients with high versus low/no mutations in tumor and ctDNA at baseline

For the association between mutation abundance and 6-month survival (Fig. 3), patients with high *KRAS* mutation abundance in tumor significantly more often had an OS < 6 months (*P* = 0.046), and those with high *KRAS* mutation abundance in ctDNA significantly more frequently had an OS < 6 months (*P* < 0.001) and a PFS < 6 months (*P* = 0.027). High *TP53* abundance in ctDNA was linked to a larger proportion of cases with an OS < 6 months (*P* < 0.001). High *CDKN2A* abundance in ctDNA was associated with more frequent PFS < 6 months (*P* = 0.048). Cases with high *SMAD4* abundance in ctDNA had more often OS < 6 months (*P* = 0.007). Patients with high *ARID1A* abundance in both tumor (*P* = 0.007) and blood (*P* = 0.040) had more frequently OS < 6 months.

**Fig. 4:**
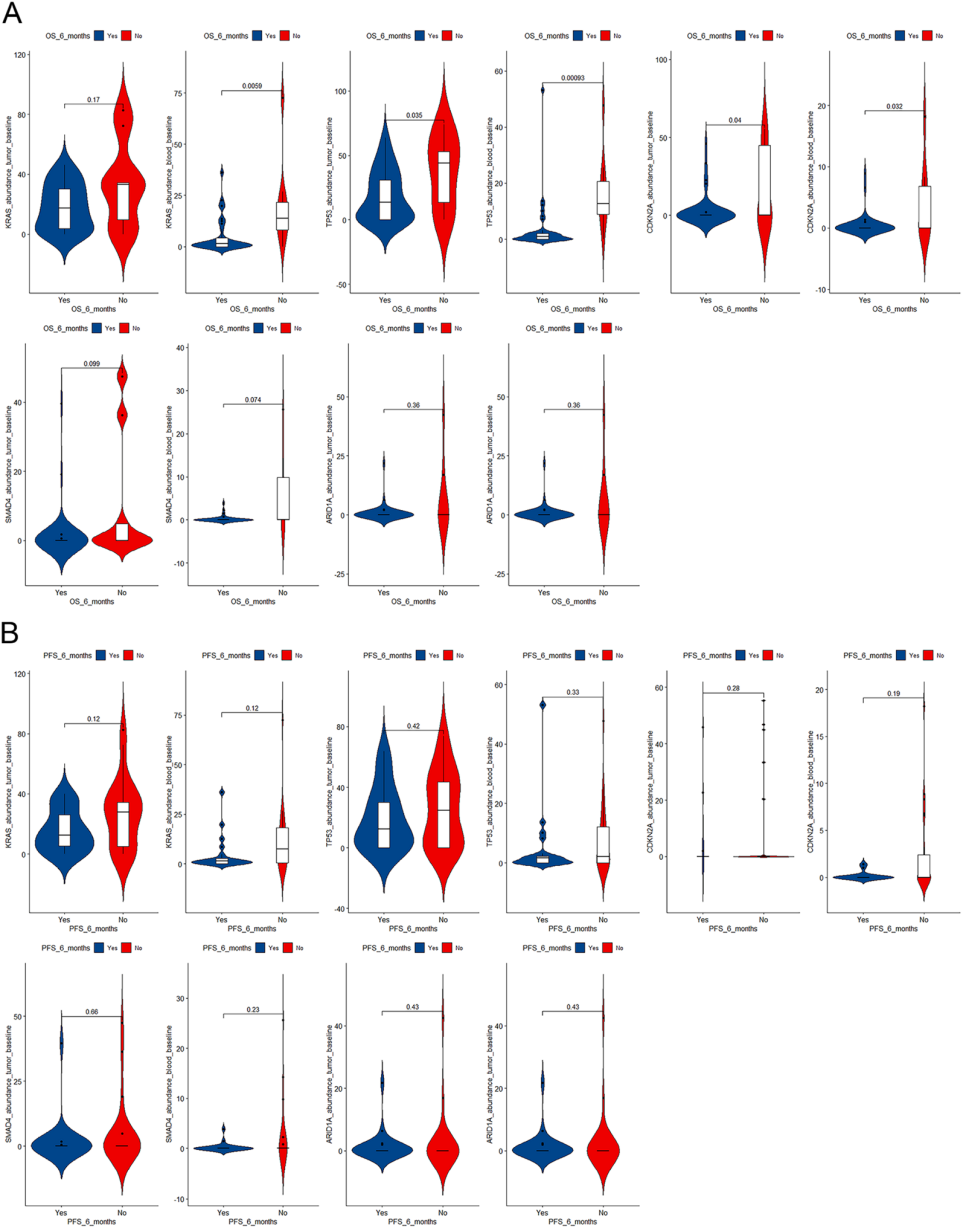
Differences in mutation abundances in tumor and ctDNA at baseline between patients with overall survival (**A**) and progression-free survival (**B**) < versus ≥ 6 months

Distributions of high versus low/no mutations of the 5 most often mutated genes with descending OS and PFS are shown in Additional file 1: Fig. S1. Patterns of the differences between patients surviving ≥ versus < 6 months were in agreement with the above findings (Fig. 4). For instance, cases with OS < 6 months significantly had a higher *KRAS* mutation abundance in ctDNA (*P* = 0.006), higher *TP53* abundances in both tumor (*P* = 0.035) and blood (*P* < 0.001), and higher *CDKN2A* abundances in both tumor (*P* = 0.040) and blood (*P* = 0.032).

**Fig. 5:**
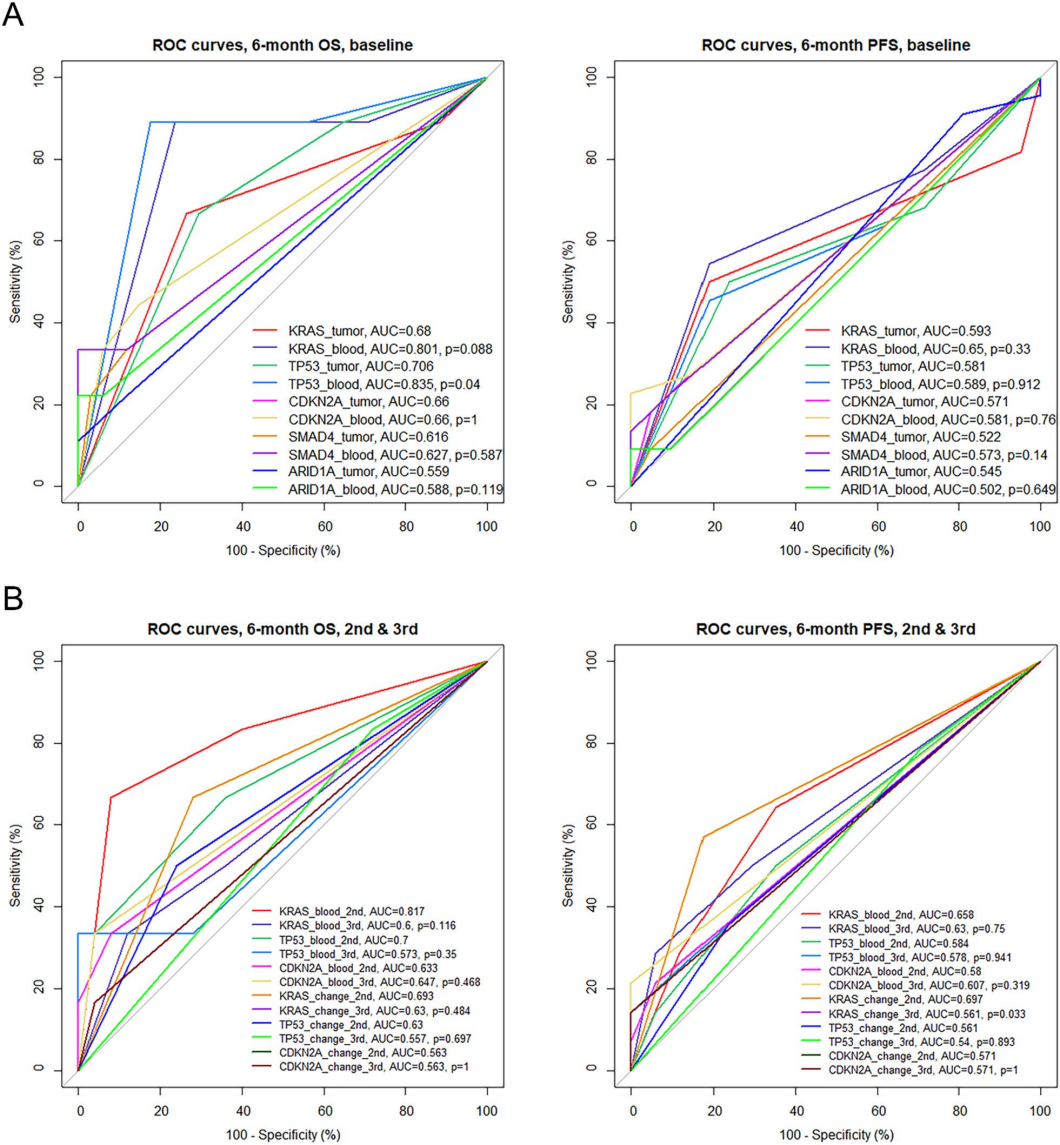
Receiver operating characteristics (ROC) curves for baseline mutations in tumor and ctDNA (**A**) and longitudinal measurements (**B**) in predicting 6-month overall survival (OS; left) and progression-free survival (PFS; right). AUC, area under the curve

**Fig. 6:**
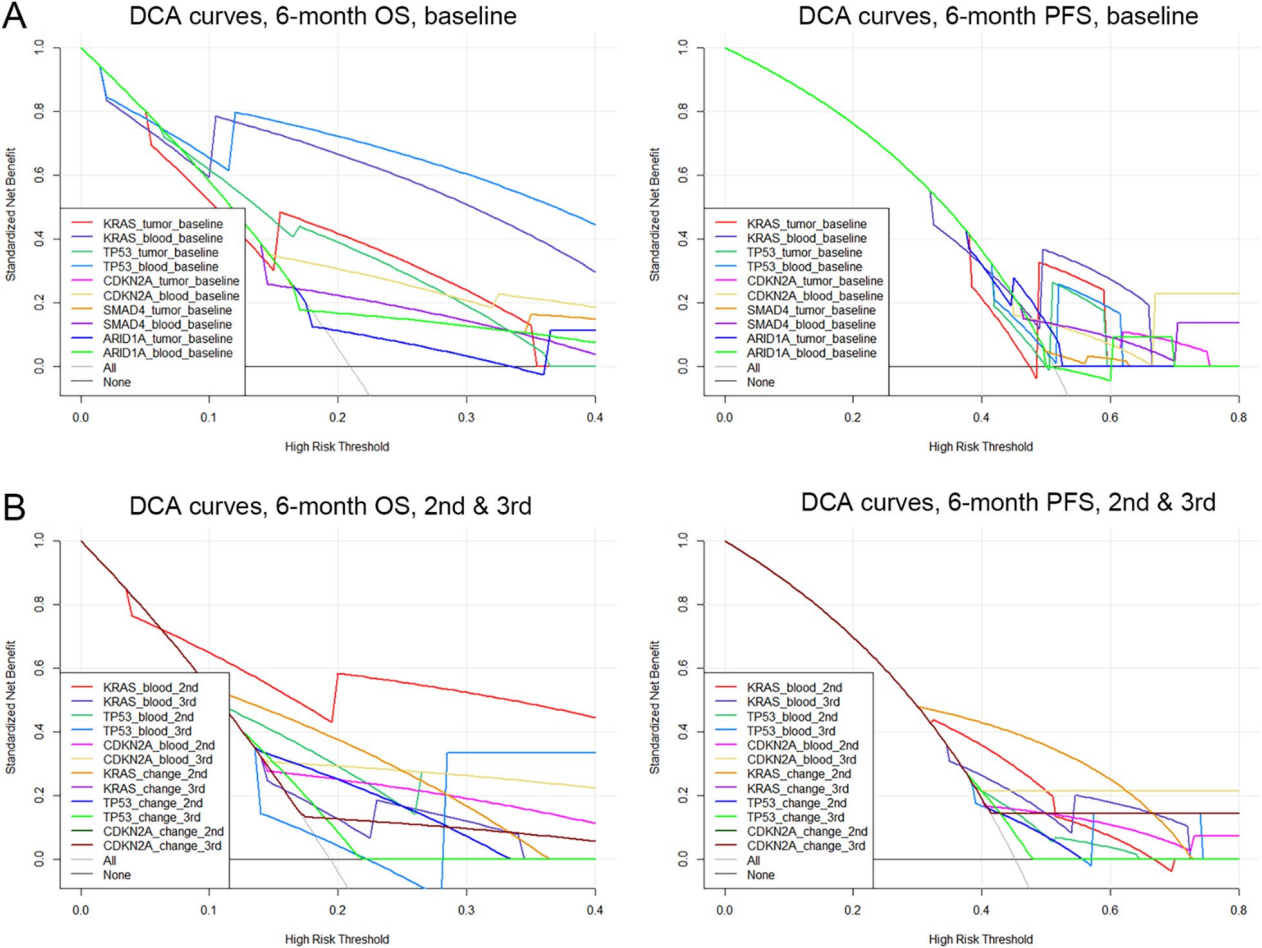
Decision curve analysis (DCA) for baseline mutations in tumor and ctDNA (**A**) and longitudinal measurements (**B**) in predicting 6-month overall survival (OS; left) and progression-free survival (PFS; right). The horizontal axis of the DCA curve is the threshold probability. The vertical axis is the net benefit after the benefit is subtracted from the harm

Mutations in tumor and blood well predicted 6-month survival especially 6-month OS (Fig. 5). For 6-month OS, the area under the curve (AUC) of the 5 most frequently mutated genes ranged from 0.588 to 0.835 when blood samples were examined, and from 0.559 to 0.706 when tumor samples were examined. Notably, the AUC for *TP53* was significantly larger when using blood than tumor samples (0.835 vs 0.706, *P* = 0.040), and the AUCs for KRAS (0.801) and TP53 (0.835) in ctDNA were higher than for the routine clinicopathologic characteristics (0.511–0.675; Additional file 1: Fig. S2). Regarding 6-month PFS, the AUC of the 5 most frequently mutated genes ranged from 0.502 to 0.650 when blood samples were examined, and from 0.522 to 0.590 when tumor samples were examined. The DCA curves for the studied genes are shown in Fig. 6.

Through multivariable analysis (Table 4), high mutation abundances of *KRAS* in both tumor (*P* = 0.007) and blood (*P* < 0.001), and high mutation abundance of *TP53* (*P* = 0.033) and *SMAD4* (*P* = 0.015) in ctDNA were significantly and independently associated with poorer OS, and high mutation abundance of *CDKN2A* (*P* = 0.018) and *SMAD4* (*P* = 0.020) in ctDNA were significantly and independently associated with poorer PFS. After internal validation by bootstraps of the Cox proportional regression analyses, the C-indexes ranged from 0.816 to 0.865 for OS and from 0.858 to 0.873 for PFS (Table 4). Associations of mutations versus no mutations in ctDNA with survival were rarely significant (data not shown).

**Table 4:** Associations of high versus low/no mutations in tumor and blood samples with survival, using multivariable-adjusted Cox proportional hazards regression

	Overall survival			Progression-free survival		
	HR (95% CI)	C-index (95% CI)	*P*	HR (95% CI)	*P*	C-index (95% CI)
*Baseline*						
* KRAS* in tumor	50.81 (2.89–894.46)	0.865 (0.791–0.939)	**0.007**	2.62 (0.61–11.21)	0.194	0.871 (0.804–0.938)
* KRAS* in blood	5281.33 (49.98–558,121.20)	0.864 (0.796–0.932)	**< 0.001**	6.11 (0.68–54.88)	0.106	0.866 (0.797–0.935)
* TP53* in tumor	5.12 (0.78–33.84)	0.828 (0.739–0.917)	0.090	1.00 (0.31–3.20)	0.999	0.858 (0.782–0.934)
* TP53* in blood	33.58 (1.33–849.52)	0.833 (0.751–0.915)	**0.033**	3.02 (0.43–21.23)	0.266	0.859 (0.785–0.933)
* CDKN2A* in tumor	0.76 (0.05–12.25)	0.816 (0.718–0.914)	0.849	0.63 (0.06–6.77)	0.701	0.859 (0.786–0.932)
* CDKN2A* in blood	3.84 (0.23–64.48)	0.822 (0.722–0.922)	0.350	12.82 (1.55–106.11)	**0.018**	0.873 (0.799–0.947)
* SMAD4* in tumor	1.33 (0.18–9.92)	0.818 (0.732–0.904)	0.779	0.69 (0.10–4.72)	0.706	0.862 (0.784–0.940)
* SMAD4* in blood	127.19 (2.60–6211.68)	0.851 (0.754–0.948)	**0.015**	34.59 (1.76–680.21)	**0.020**	0.868 (0.783–0.953)
* ARID1A* in tumor	NE			NE		
* ARID1A* in blood	20.11 (0.78–521.34)	0.846 (0.729–0.963)	0.071	4.97 (0.30–83.58)	0.266	0.858 (0.778–0.938)
*2nd measurement*						
* KRAS* in blood	180.62 (0.05–657561.60)	0.865 (0.830–0.900)	0.214	2.74 (< 0.01–20.56)	0.557	0.874 (0.827–0.921)
* TP53* in blood	1.28 (0.01–217.63)	0.862 (0.839–0.885)	0.925	0.05 (< 0.01–2.65)	0.138	0.882 (0.863–0.901)
* CDKN2A* in blood	140.24 (0.03–790450.30)	0.868 (0.830–0.906)	0.262	359.82 (0.03–4515432.00)	0.222	0.867 (0.825–0.909)
*3rd measurement*						
* KRAS* in blood	7.41 (0.42–132.48)	0.850 (0.823–0.877)	0.173	2.73 (0.40–18.66)	0.306	0.879 (0.826–0.932)
* TP53* in blood	4.72 (< 0.01–5037.12)	0.845 (0.817–0.873)	0.663	3.02 (0.03–301.50)	0.638	0.872 (0.844–0.900)
* CDKN2A* in blood	NE			NE		

Bold values indicate statistical significance (*P* < 0.05)

Associations of mutations in tumor and ctDNA with OS and PFS were then explored using multivariable-adjusted Cox proportional hazards regression, with hazard ratios (HRs) and corresponding 95% confidence intervals (CIs) computed. The adjusted factors included sex, age, ECOG performance status, tumor differentiation grade, primary tumor location, liver metastasis, lung metastasis, bone metastasis, distant lymph node metastasis, CA19-9 level, CEA level, CA125 level, cycle of chemotherapy, and S-1 maintenance therapy. C-indexes and the corresponding 95% CIs were computed for internal validation by bootstraps of the Cox proportional regression models (number of bootstrap replicates = 200)

*HR* hazard ratio, *CI* confidence interval; *NE* not estimable

### Associations of repeated measurements of gene mutations in ctDNA with efficacy of NPS

In the 31 patients with repeatedly collected blood samples (Additional file 1: Fig. S3), high *KRAS* mutation abundance in ctDNA at the second measurement remained linked to worse OS (*P* = 0.019), and high *KRAS* mutation abundance in ctDNA at the third measurement was still associated with worse OS (*P* = 0.003) and poorer PFS (*P* = 0.014). Patients with high *TP53* mutation abundance in ctDNA at the third measurement (OS, *P* < 0.001; PFS, *P* < 0.001) and those with high *CDKN2A* mutation abundance in ctDNA at the second measurement (OS, *P* = 0.004; PFS, *P* = 0.018) significantly had worse survival.

For the association between mutation abundance in ctDNA and 6-month survival (Additional file 1: Fig. S4), patients with high *KRAS* mutation abundance at the second measurement (*P* = 0.012) and those with high *TP53* mutation abundance at the third measurement (*P* = 0.040) significantly more often had an OS < 6 months. Differences in mutations in ctDNA between patients surviving ≥ versus < 6 months are illustrated in Additional file 1: Fig. S5. Cases with OS < 6 months significantly had a higher *TP53* mutation abundance (*P* = 0.020).

Regarding the 3 most frequently mutated genes in ctDNA (*KRAS*, *TP53*, and *CDKN2A*; Fig. 4), the AUCs for 6-month OS ranged from 0.633 to 0.817 for the second measurement, and from 0.573 to 0.647 for the third measurement, and the AUCs for 6-month PFS ranged from 0.580 to 0.658 for the second measurement, and from 0.578 to 0.630 for the third measurement. After internal validation by bootstraps of the Cox proportional regression analyses, the C-indexes ranged from 0.845 to 0.868 for OS and from 0.867 to 0.882 for PFS (Table 4).

Through longitudinal analyses (Table 5), repeated measurements of *KRAS* mutation in ctDNA for 2 and 3 times significantly differentiated patients with OS ≥ 6 or ≥ 12 months and those with PFS ≥ 6 months versus their counterparts, and measurement for 2 times also significantly identified patients with PD as best objective response. Examinations of TP53 for 2 times significantly screened patients with OS ≥ 6 months and those with PD as best objective response, and assessments for 3 times significantly identified cases with OS ≥ 6 months and those with PR as best objective response. Repeated measurement of *CDKN2A* for 2 or 3 times significantly differentiated patients with OS ≥ 6 or ≥ 12 months and those with PFS ≥ 6 months against their counterparts.

**Table 5:** Longitudinal data analysis of survival outcomes by *KRAS*, *TP53*, and *CDKN2A* mutations

Gene	Measured time	OS ≥ 6 months	OS ≥ 12 months	PFS ≥ 6 months	PFS ≥ 12 months	PR as BOR^a^	PD as BOR^a^
		*P* value	*P* value	*P* value	*P* value	*P* value	*P* value
*KRAS*	2	**0.001**	**0.026**	**0.021**	0.178	0.344	**0.012**
	3	**0.014**	**0.002**	**0.002**	0.057	0.315	0.681
*TP53*	2	**< 0.001**	0.083	0.059	0.151	0.108	**0.015**
	3	**0.001**	0.149	0.098	0.184	**0.044**	0.164
*CDKN2A*	2	**0.027**	**0.027**	**0.004**	0.465	0.202	NE
	3	**0.031**	**0.031**	**0.002**	0.439	0.176	NE

Bold values indicate statistical significance (*P* < 0.05)

*OS* overall survival, *PFS* progression-free survival, *PR* partial response, *PD* progressive disease, *BOR* best objective response, *NE* not estimable

^a^During first-line chemotherapy

### Clinical assessment of the combined gene panel

Notably, 48% of the patients had a ctDNA progression earlier than radiologic progression, with a median lead time of 60 days, and 35% of the patients had a ctDNA progression on the same day with radiologic progression. 42% of the patients had a ctDNA progression earlier than CA19-9 progression, with a median lead time of 58 days, and 48% of the patients had a ctDNA progression on the same day with CA19-9 progression. (Table 6).

**Table 6:** Comparisons between ctDNA progression, radiologic progression, and CA19-9 progression

Comparison	Value
ctDNA progression vs radiologic progression	
ctDNA progression earlier	15 (48.4)
Lead time (days)	60 (28–94)
On the same day	11 (35.5)
Radiologic progression earlier	5 (16.1)
ctDNA progression vs CA19-9 progression	
ctDNA progression earlier	13 (41.9)
Lead time (days)	58 (44–93)
On the same day	15 (48.4)
CA19-9 progression earlier	3 (9.7)

Continuous variables are shown as median (interquartile range), and categorical variables as count (percentage [%])

### Survival of patients with inconsistencies between ctDNA and tumor mutations

**Fig. 7:**
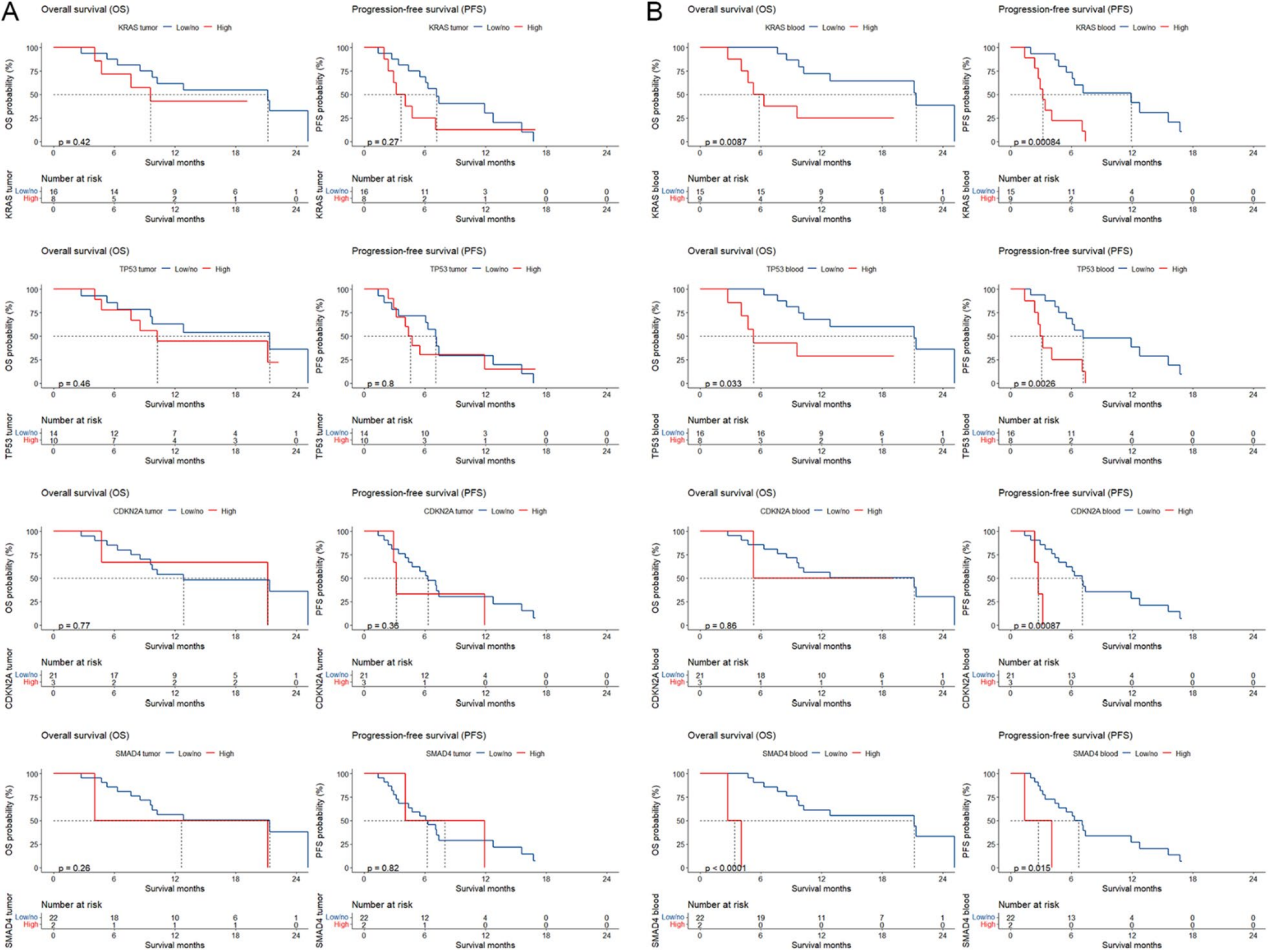
Kaplan–Meier plots for associations of mutations in tumor (**A**) and ctDNA (**B**) at baseline with overall survival and progression-free survival

At baseline, 24 patients had inconsistencies between mutations of any of the 5 most commonly mutated genes (*KRAS*, *TP53*, *CDKN2A*, *SMAD4*, and *ARID1A*) in ctDNA and tumor (Fig. 1C). Among them, high versus low/no mutation of *KRAS*, *TP53*, *CDKN2A*, or *SMAD4* in tumor was not significantly associated with OS or PFS; however, compared with low/no mutation, high mutation of *KRAS* (OS, *P* = 0.009; PFS, *P* = 0.001), *TP53* (OS, *P* = 0.033; PFS, *P* = 0.003), *CDKN2A* (PFS, *P* = 0.001), or *SMAD4* (OS, *P* < 0.001; PFS, *P* = 0.015) in ctDNA was significantly linked to better survival (Fig. 7), suggesting greater predictive values of ctDNA mutations for such patients.

**Fig. 8:**
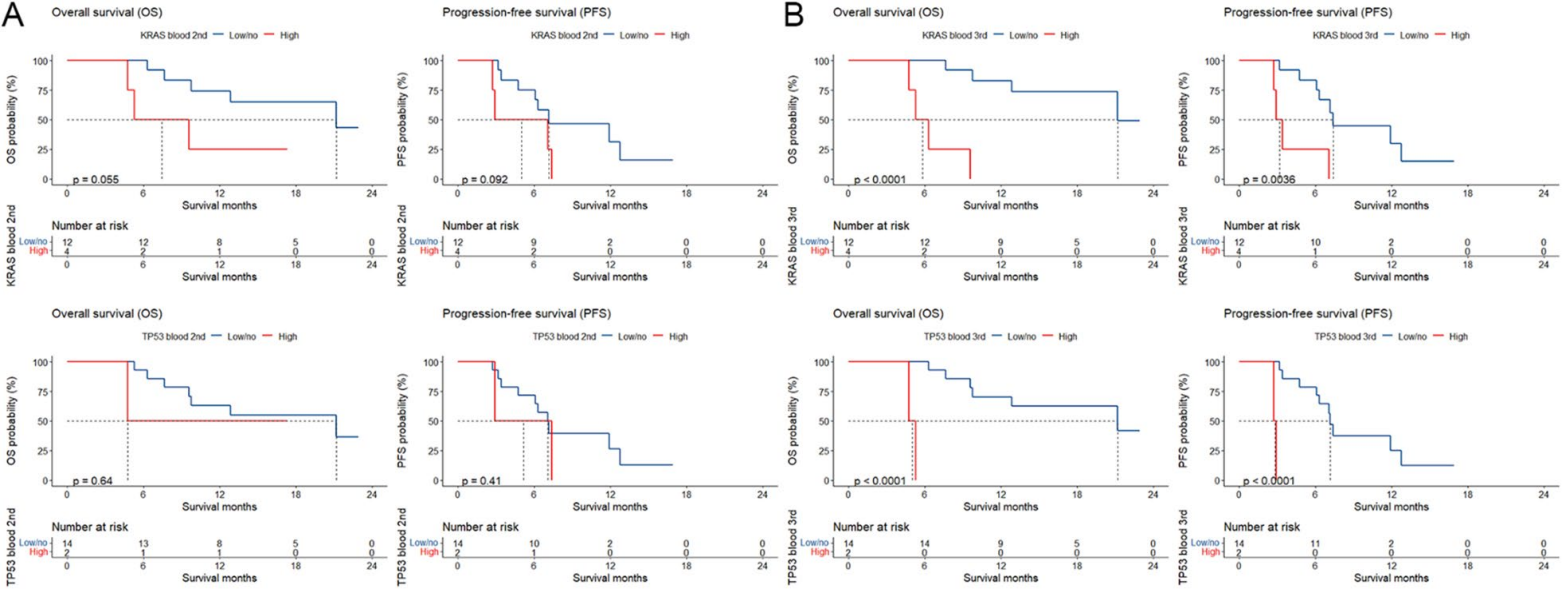
Kaplan–Meier plots for associations of mutations in ctDNA at the second (**A**) and third (**B**) measurements with overall survival and progression-free survival

16 patients with inconsistencies between ctDNA and tumor mutations had repeated measurements of ctDNA mutations. Among them, high versus low/no *KRAS* or *TP53* mutation in ctDNA at the second measurement was not significantly associated with OS or PFS; however, at the third measurement, high mutation of *KRAS* (OS, *P* < 0.001; PFS, *P* = 0.004) or *TP53* (OS, *P* < 0.001; PFS, *P* < 0.001) in ctDNA was significantly linked to better survival compared with low/no mutation (Fig. 8).

## Discussion

In this study focusing on the value of mutations in ctDNA in predicting response of mPDAC to the NPS regimen, we first revealed the frequencies of mutation and high mutation of the most commonly mutated genes and uncovered the inter-correlations of mutation abundances between tumor and blood samples. We then showed the temporal changes of the proportions of mutations and high mutations and their links to best objective response. We further showed the univariable and multivariable associations of high mutations in ctDNA and tumor with survival, both at baseline and after repeated measurements, quantified the predictive performance for 6-months survival, and confirmed the findings using longitudinal data analyses. Specifically, we computed the lead time of ctDNA progression over radiologic or CA19-9 progression. Our findings suggest that high mutations of driving genes in ctDNA and their temporal changes can be helpful to predict efficacy of NPS chemotherapy in patients with mPDAC, as compared to the rather limited prognostic significances of mutations versus non-mutations [18]. We used 425-gene NGS, while droplet digital PCR (ddPCR) testing *KRAS* mutation was most commonly utilized in previous studies.

During cancer progression, ctDNA most often originates from small extracellular vesicles in PDAC, and suggests tumor burden [35, 36]. For resected PDAC, preoperative and postoperative ctDNA examinations focusing on *KRAS* mutations are significantly and strongly prognostic [37–40]. Mutation load in ctDNA typically increases with more advanced disease stage [41]. In a previous study [42], ctDNA was detectable in all treatment-naïve patients with metastatic PDAC, which is in agreement with our study. We found that the most commonly mutated somatic genes in tumor tissue and ctDNA in patients with mPDAC included *KRAS*, *TP53*, *CDKN2A*, *SMAD4*, and *ARID1A*, with a median somatic mutation number of 5 and 3 in tumor and blood samples, respectively, which is agreed by previous studies [41, 43, 44].

KRAS is a key driving oncogene in PDAC [45]. We found that high abundances of *KRAS* mutations in ctDNA at baseline and the second and third measurements were significantly and independently associated with poorer OS and PFS among patients with mPDAC receiving NPS chemotherapy. Multiple previous studies [31, 46–54] supported that mutated *KRAS* in ctDNA predicted poorer survival in patients receiving chemotherapy, despite the different regimens used. *KRAS-G12D* mutation, which took up 51% of all cases in our study, is linked to even shorter survival than *KRAS-G12V* and other mutations [55]. We further found that *KRAS* mutation rate in ctDNA dropped from 74% at baseline to 48% at the second measurement and 39% at the third measurement, which is consistent with a previous study [56] on stage IV PDAC showing that *KRAS* mutation rates were 91% and 45% before and during chemotherapy, respectively. In our study, *KRAS* mutation in ctDNA was more prevalent in patients with liver metastasis (82%), which was supported by previous studies [52, 56–58]. A Japanese study [59] reported that KRAS mutation appeared concurrently with liver metastasis. We also found that KRAS mutation in ctDNA was significantly higher in patients with CA19-9 ≥ 2000 U/mL (92%), which is supported by previous studies [60, 61]. We further found that for predicting 6-month OS, *KRAS* and *TP53* mutations in ctDNA had higher AUCs than the other clinicopathologic characteristics, and previous studies [56, 62] also showed that ctDNA was more accurate for monitoring chemotherapy efficacy than CA19-9, the major prognostic circulating tumor marker for PDAC.

The role of TP53 has been well characterized in PDAC, and TP53 mutation is also a major driver of PDAC and has been connected to treatment resistance and poor prognosis. We previously found that TP53 was associated with the tumor immune microenvironment (TIME) in PDAC [20]. In this study, we found that in patients with mPDAC TP53 mutations in ctDNA at initial diagnosis and the third measurement but not in tumor predicted shorter OS, both in univariable and multivariable analyses, which is in line with a previous study [63]. *TP53*-mutated ctDNA at baseline predicted early tumor progression in patients with PDAC receiving FOLFIRINOX chemotherapy [64]. Through both univariable and multivariable analyses, we also revealed that high CDKN2A and SMAD4 mutation abundances in ctDNA but not in tumor and high ARID1A mutation abundances in both ctDNA and tumor at baseline and/or the second measurement were linked to inferior OS and/or PFS. Pathogenic variants in CDKN2A increase the risk for pancreatic cancer (~ 5% to 24% lifetime risk), and individuals with pathogenic variants in CDKN2A tend to have an earlier onset of cancer [65, 66]. SMAD4, a transforming growth factor (TGF)-β/BMP signaling effector and a tumor suppressor, is frequently mutated in PDAC and actively participates in the interaction between cancer and stromal myeloid cells and mediates the response of cancer cells to stromal chemokine [67]. It drives distal dissemination and its loss is associated with and poor prognosis in PDAC [68–70]. ARID1A-deficient undifferentiated carcinoma exhibited cellular discohesion and rhabdoid morphology [71]. Mutation surveillance of these genes could effectively monitor response of mPDAC to NPS chemotherapy.

We found that, the correlation coefficient *r* and the *κ* value for the concordance between mutations of the 5 most frequently mutated genes in matched tumor and blood samples ranged from 0.49 to 0.93 and from 0.48 to 0.76, respectively, which is in line with previous studies [41, 64]. This suggests that mutations in ctDNA could well reflect and serve as surrogate for the mutations in the deriving tumor. We further showed that, for predicting 6-month OS, the AUCs for the 5 most frequently mutated genes ranged from 0.59 to 0.84 in ctDNA compared to 0.56 to 0.71 in tumor, and the AUC for TP53 was significantly larger in ctDNA than in tumor. Particularly, the AUCs for KRAS (0.80) and TP53 (0.84) were significantly larger than routine patient and tumor characteristics (0.51–0.68). Thus, examining the specific mutations in ctDNA obtained from peripheral blood, an easily obtainable sample, could effectively and accurately predict the survival outcomes in patients receiving NPS chemotherapy, thus guiding the optimal utilization of this promising combination.

Dynamics of mutation abundance in ctDNA may correlate with tumor burden after chemotherapy and suggest treatment response [36, 72]. We found that after chemotherapy initiation, mutation abundances in ctDNA mostly first decreased before increasing with disease progression, and that persistence of high mutation abundances of *KRAS*, *TP53*, and *CDKN2A* was linked to poorer survival; these are supported by previous studies [51, 73, 74]. Previous studies [42, 59, 75, 76] supported that trends of mutation abundance in ctDNA were consistent with changes of CA19-9 and clinically reported disease burden. Abundance of mutations in ctDNA declines in chemotherapy-responding patients, with increased abundance observed at disease progression in cases resistant to chemotherapy [36, 72]. Through dynamic monitoring of the abundance changes of gene mutations in ctDNA in blood, we found that a significant early decrease in *KRAS* and/or *TP53* ctDNA abundance effectively predicted response of NPS regimen. Longitudinal assessments of KRAS-mutated ctDNA can correctly predict > 80% of patient responses [61]. In our study, longitudinal data analyses confirmed that repeated measurements of KRAS, TP53, and CDKN2A for 2 or 3 times could significantly identify patients with longer survival and better objective response. We found that patients who showed an unfavorable trend of mutation abundances of KRAS, TP53, and/or CDKN2A at the second or third measurement mostly experienced PD within 4 months of chemotherapy. Cases with an obvious decrease in mutation abundance ≥ 2% mostly achieved PR as best response. Interestingly, mutation abundances at each measurement appeared more predictive of treatment response than changes in abundances [77]. A Japanese study [78] also supported that presence of KRAS-mutated ctDNA at baseline had greater impact on therapeutic benefits than the changes.

We found that, using longitudinal analyses of combined mutations of *KRAS*, *TP53*, *CDKN2A*, *SMAD4*, and *ARID1A*, 48% of the patients had a ctDNA progression earlier than radiologic progression, and 42% a ctDNA progression earlier than CA19-9 progression, both with a median lead time of about 2 months, which is supported by previous reports [46, 79]. In resected PDAC, ctDNA could predict recurrence with a median lead time of 84 days. [73] Another US study [80] reported that ctDNA recurrence was 6.5 months ahead radiologic relapse after resection. These suggest that in a considerable proportion of cases with PDAC, mutations detection in ctDNA could effectively predict disease progression and treatment resistance months ahead clinical cancer progression, allowing for abundance time for adjustment of treatments regimens and/or surveillance schedules. Comprehensive evaluations integrating ctDNA, imaging, and CA19-9 analyses could promisingly identify progressive disease during NPS chemotherapy at the earliest.

As of now, FOLFIRINOX or gemcitabine in combination with albumin-bound paclitaxel represents the mainstream chemotherapy for advanced/metastatic pancreatic cancer. van der Sijde et al. [64] reported that in 48 patients with PDACs of all stages, *TP53* ctDNA mutation before FOLFIRINOX was linked to early tumor progression in multivariable analysis. Wei et al. [36] showed that in 38 patients with advanced PDAC receiving first-line FOLFIRINOX treatment, the mutant allele fraction for altered loci in ctDNA before treatment correlated with cancer stage, metastatic burden, and OS. In the 17 patients with serial blood samples collected after FOLFIRINOX chemotherapy, allele fraction for specific altered loci declined in chemotherapy-responding cases, but increased at the time of disease progression in cases resistant to FOLFIRINOX. The dynamics of total ctDNA concentration correlated with tumor burden following FOLFIRINOX chemotherapy. Tjensvoll et al. [76] revealed that in 14 patients with advanced pancreatic cancer receiving gemcitabine or FOLFIRINOX, positive *KRAS*-mutated ctDNA at baseline was significantly linked to disease progression and survival. Del Re et al. [81] showed that in 27 patients with advanced PDAC receiving first-line FOLFIRINOX or gemcitabine plus nab-paclitaxel, an increase in *KRAS*-mutant ctDNA 15 days after treatment initiation was significantly associated with shorter PFS and OS.

Motobayashi et al. [82] reported that in 18 patients with locally advanced or metastatic pancreatic cancer receiving first-line gemcitabine plus nab-paclitaxel, an increase in the mutant allele frequency of *KRAS*-mutated ctDNA from Day 0 to 7 after chemotherapy initiation was significantly linked to disease progression and shorter PFS; however, positive pretreatment ctDNA status was not associated with disease progression. Dayimu et al. [48] showed that in patients with metastatic PDAC receiving gemcitabine plus nab-paclitaxel, positive *KRAS*-mutated ctDNA correlated with worse OS after multivariable adjustment; however, the association of longitudinal evaluation of *KRAS*-mutated ctDNA with OS was not significant. In the CCTG PA.7 phase II trial [51] of gemcitabine plus nab-paclitaxel as initial therapy for metastatic PDAC, survival was significantly longer for patients with KRAS-wildtype ctDNA.

In this prospective study specifically focusing on patients with metastatic PDAC receiving NPS treatment, we adopted novel sensitive thresholds for ctDNA and first analyzed the significance of mutations with high abundance versus those with low/no abundance, and the findings might not be easily integrated with previous researches [36, 48, 51, 64, 76, 81–84] mostly analyzing mutation versus non-mutation in ctDNA and/or focusing on patients with resected PDAC receiving neoadjuvant and/or adjuvant chemotherapy. Few studies had a study design similar to ours. The significance of ctDNA monitoring in selecting the best chemotherapy regimen for metastatic PDAC needs to be addressed in future studies.

This single-center study was majorly limited by the limited case number. Larger-scale multicenter investigations are needed to validate the intriguing findings. Nevertheless, this study offers important hints for precisely predict clinical responses of mPDAC to NPS chemotherapy, with the potential to effectively guide clinical utilization of the active combination regimen.

**Fig. 9:**
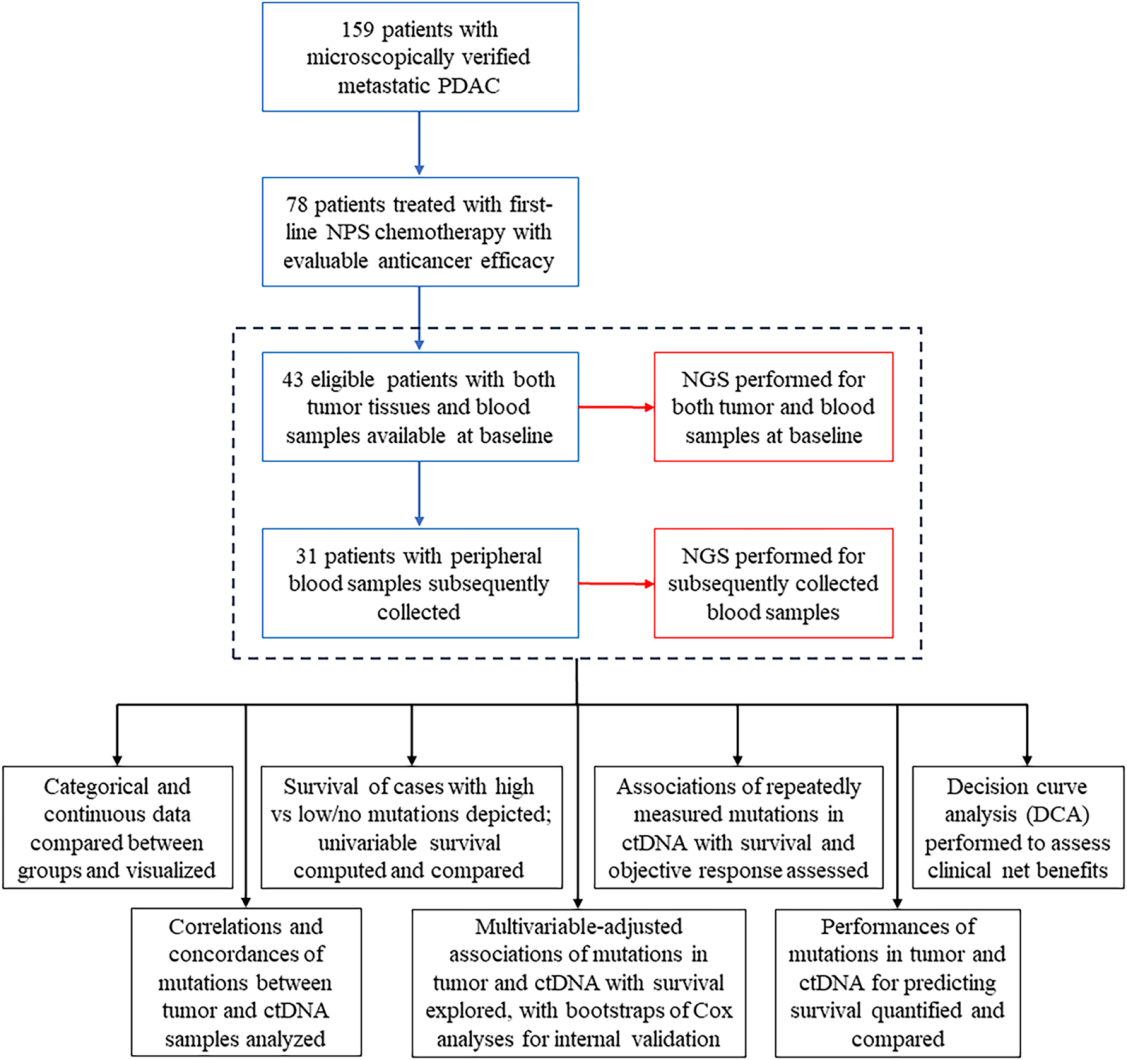
Remark diagram of this study

Conclusively, in this prospective study (Fig. 9), high mutations of multiple driving genes (e.g., KRAS, TP53, and SMAD4) and their dynamic changes in ctDNA extracted from easily obtainable peripheral blood could effectively predict response of mPDAC to NPS chemotherapy, with good predictive performance superior to routine clinicopathologic parameters. Inspiringly, longitudinal ctDNA tracking could predict disease progression about 2 months ahead of radiologic or CA19-9 evaluations, with the potential to precisely devise individualized therapeutic strategies for mPDAC.

### Supplementary Information

**Additional file 1: Table S1.** Mutated genes at baseline. **Figure S1.** Distributions of high versus low/no mutations with decreasing overall survival (**A**) and progression-free survival (**B**). **Figure S2.** Receiver operating characteristics (ROC) curves for baseline patient and tumor characteristics in predicting 6-month overall survival (OS) and progression-free survival (PFS). AUC, area under the curve. **Figure S3.** Kaplan-Meier plots for associations of mutations in ctDNA at repeated measurements with overall survival (**A**) and progression-free survival (**B**). **Figure S4.** Proportions of cases with overall survival (OS; **A**) and progression-free survival (PFS; **B**) < versus ≥ 6 months in patients with high versus low/no mutations in ctDNA at repeated measurements. **Figure S5.** Differences in mutation abundances in ctDNA at repeated measurements between patients with overall survival (**A**) and progression-free survival (**B**) < versus ≥ 6 months.

## Data Availability

Restrictions apply to the availability of the data for this study, which were used under license, and so are not publicly available.
