# Supplementary material for: High somatic mutations in circulating tumor DNA predict response of metastatic pancreatic ductal adenocarcinoma to first-line nab-paclitaxel plus S-1: prospective study
Source: J Transl Med. 2024 Feb 20;22:184. doi: 10.1186/s12967-024-04989-z (PMC10877900; doi:10.1186/s12967-024-04989-z)
Supplement: Supplementary file 1 — Additional file 1: Table S1. Mutated genes at baseline. Figure S1. Distributions of high versus low/no mutations with decreasing overall survival (A) and progression-free survival (B). Figure S2. Receiver operating characteristics (ROC) curves for baseline patient and tumor characteristics in predicting 6-month overall survival (OS) and progression-free survival (PFS). AUC, area under the curve. Figure S3. Kaplan-Meier plots for associations of mutations in ctDNA at repeated measurements with overall survival (A) and progression-free survival (B). Figure S4. Proportions of cases with overall survival (OS; A) and progression-free survival (PFS; B) < versus ≥ 6 months in patients with high versus low/no mutations in ctDNA at repeated measurements. Figure S5. Differences in mutation abundances in ctDNA at repeated measurements between patients with overall survival (A) and progression-free survival (B) < versus ≥ 6 months. [file 12967_2024_4989_MOESM1_ESM.docx]

**Additional file**

**Table S1.** Mutated genes at baseline

**Figure S1.** Distributions of high versus low/no mutations with decreasing overall survival (**A**) and progression-free survival (**B**).

**Figure S2.** Receiver operating characteristics (ROC) curves for baseline patient and tumor characteristics in predicting 6-month overall survival (OS) and progression-free survival (PFS). AUC, area under the curve.

**Figure S3.** Kaplan-Meier plots for associations of mutations in ctDNA at repeated measurements with overall survival (**A**) and progression-free survival (**B**).

**Figure S4.** Proportions of cases with overall survival (OS; **A**) and progression-free survival (PFS; **B**) < versus ≥ 6 months in patients with high versus low/no mutations in ctDNA at repeated measurements.

**Figure S5.** Differences in mutation abundances in ctDNA at repeated measurements between patients with overall survival (**A**) and progression-free survival (**B**) < versus ≥ 6 months.

**Table S1.** Mutated genes at baseline

| Pseudo ID | Genes with the largest mutation abundance | Other mutated genes |
| --- | --- | --- |
| 1 | *TP53/BRAF* |  |
| 2 | *KRAS/TP53* |  |
| 3 | *KRAS/TP53/CDKN2A* | *AKT2/CCNE1* |
| 4 | *KRAS/TP53/CDK12* | *CDK12/GNAS* |
| 5 | *KRAS/ATM/HER2* | *ATM/HER2/MYCL/SOX2* |
| 6 | *KRAS/TP53/CDKN2A/SMAD4* | *RB1* |
| 7 | *NRAS 1.3* |  |
| 8 | *KRAS/TP53/CDKN2A* | *ERCC3/GATA3/RUNX1T1* |
| 9 | *KRAS/TP53/ARID1A* | *HER4/ERCC4/GNAQ* |
| 10 | *KRAS/TP53* | *FGFR1/ZNF703* |
| 11 | *KRAS/TP53* | *MYC/PGR* |
| 12 | *PALB2/CREBBP/KEAP1* | *PALB2/CREBBP/KEAP1* |
| 13 | *KRAS/TP53* |  |
| 14 | *KRAS/TP53* | *CYLD/SMARCA4* |
| 15 | *KRAS/CDKN2A* | *GNAS/BAP1* |
| 16 | *KRAS/TP53/CDKN2A/SMAD4* | *RNF43/RAD50/CCNE1* |
| 17 | *KRAS/TP53/SMAD4* | *ERBB4/SMARCB1/CDK6* |
| 18 | *KRAS/TP53* | *NFKBIA/PKHD1* |
| 19 | *KRAS/TP53* | *SMO 2.7/MAP3K1/EPHA5/ARID2* |
| 20 | *KRAS* | *VEGFA* |
| 21 | *KRAS/TP53/ARID1A* | *MAP2K2/MTOR/SMAD3/XRCC1* |
| 22 | *KRAS* | *SMAD3/CHD4* |
| 23 | *KRAS/TP53/CDKN2A* | *NOTCH2/GATA4/FGFR1/ZNF703* |
| 24 | *KRAS* |  |
| 25 | *KRAS/TP53* | *GRM8 2.1* |
| 26 | *KRAS/TP53/SMAD4* | *ABCB1* |
| 27 | *KRAS/TP53/CDKN2A* | *SMARCA4/MAP2K4* |
| 28 | *PTPN11 1.8/MET 1.1* |  |
| 29 | *KRAS/TP53* | *FGFR4/FLT4/MLH1/TB1/PDGFRB* |
| 30 | *KRAS/TP53/CDKN2A* | *GATA2* |
| 31 | *KRAS/TP53/SMAD4* | *NF1/RNF43/ZNF703/BAD* |
| 32 | *KRAS/TP53/SMAD4/ARID1A* | *BAI3/CDK6/CYP2B6/HGF/SETD2* |
| 33 | *KRAS* | *AKT3/NTRK1/MDM4/DDR2/CTNNB1/RNF43* |
| 34 | *KRAS* | *CDKN1C* |
| 35 | *KRAS/TP53* | *ATRX/MCL1* |
| 36 | *KRAS/TP53* |  |
| 37 | *KRAS/TP53* | *GATA6* |
| 38 | *KRAS/TP53* | *GATA6/MSH6/PIK3CA/PRDM1/STK11/MCL1/KDM5A* |
| 39 | *KRAS/TP53* | *TPMT/IDH1/CHD4* |
| 40 | *ARID1A* | *TSC2/TGFBR2/SMARCB1/POLE/NF1* |
| 41 | *KRAS/SMAD4/TP53/CDKN2A* | *ABCB1/ERBB3/ERCC4/GATA3* |
| 42 | *KRAS/KDM6A/ZNF703* | *KDM6A/ZNF703* |
| 43 | *KRAS/TP53/MAP2K4* | *MAP2K4* |


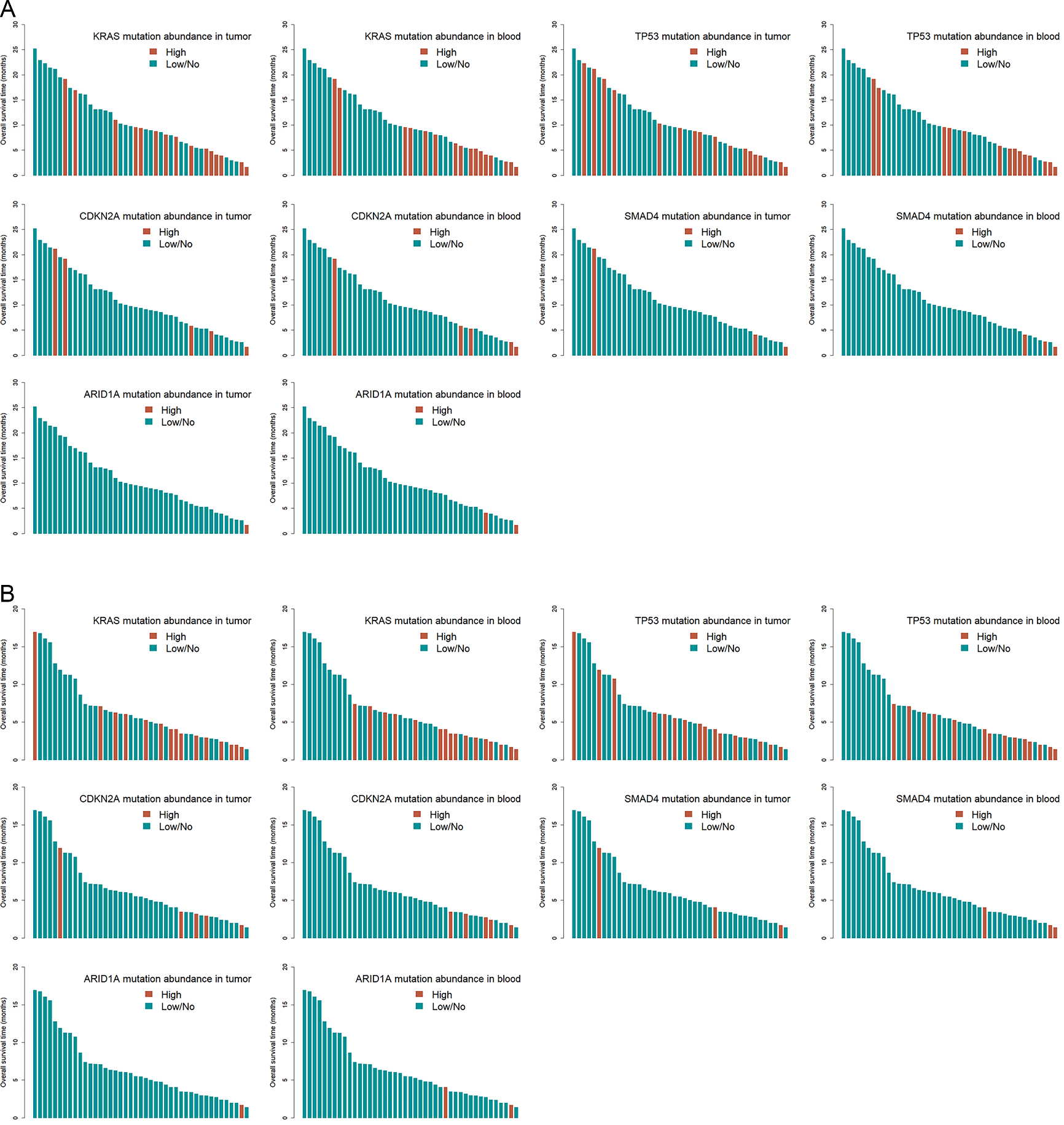


**Figure S1.** Distributions of high versus low/no mutations with decreasing overall survival (**A**) and progression-free survival (**B**).


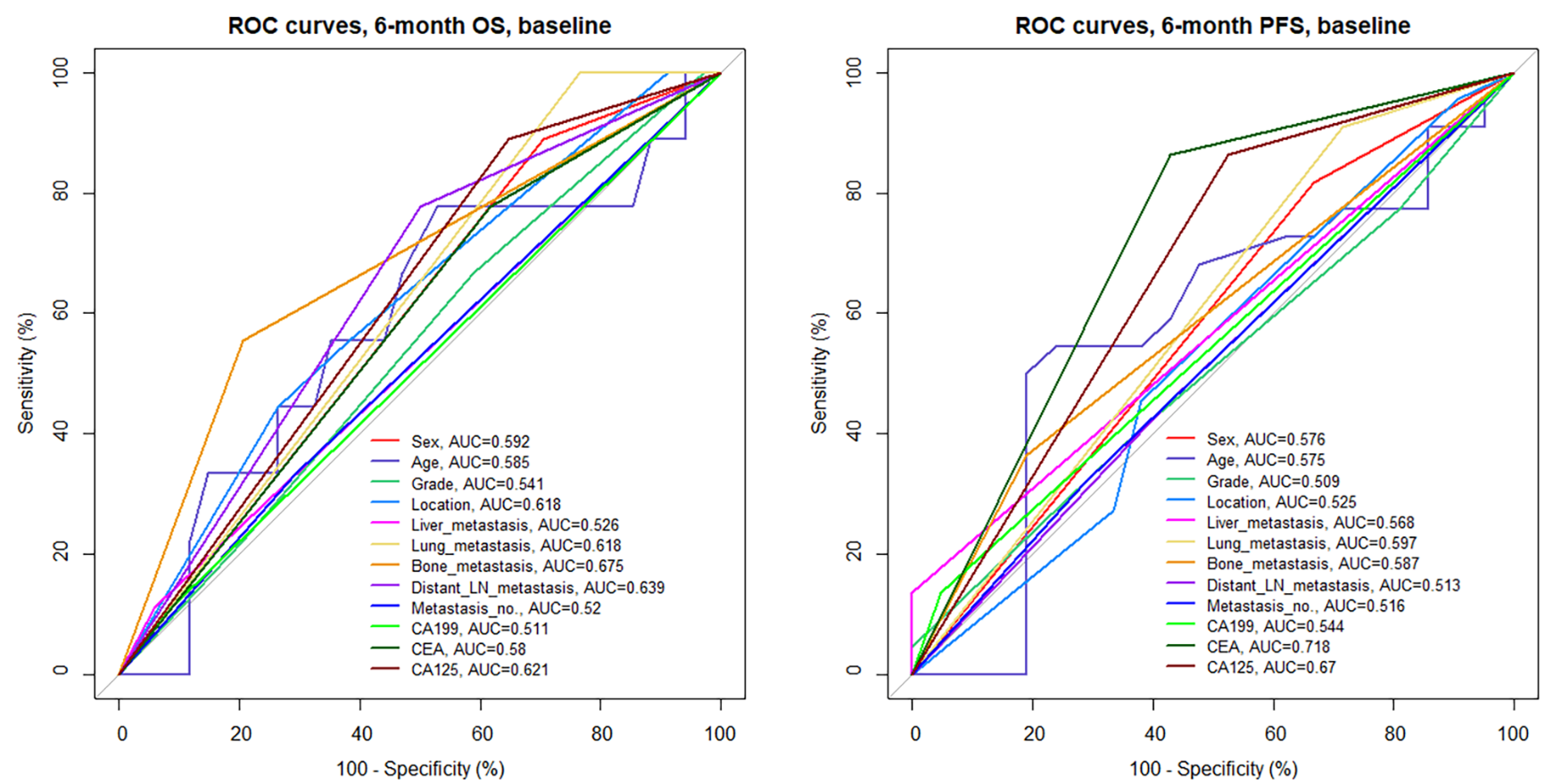


**Figure S2.** Receiver operating characteristics (ROC) curves for baseline patient and tumor characteristics in predicting 6-month overall survival (OS) and progression-free survival (PFS). AUC, area under the curve.


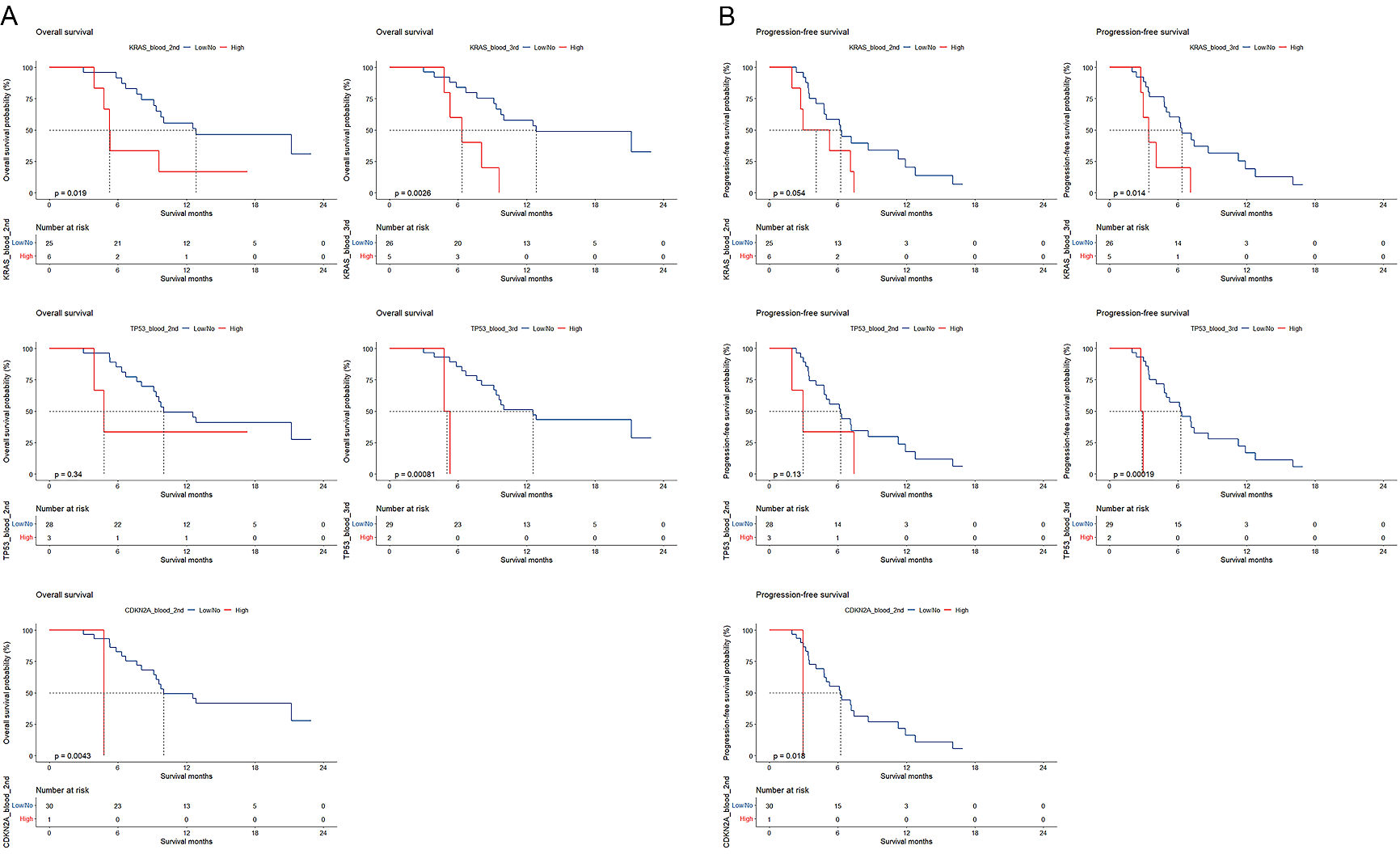


**Figure S3.** Kaplan-Meier plots for associations of mutations in ctDNA at repeated measurements with overall survival (**A**) and progression-free survival (**B**).


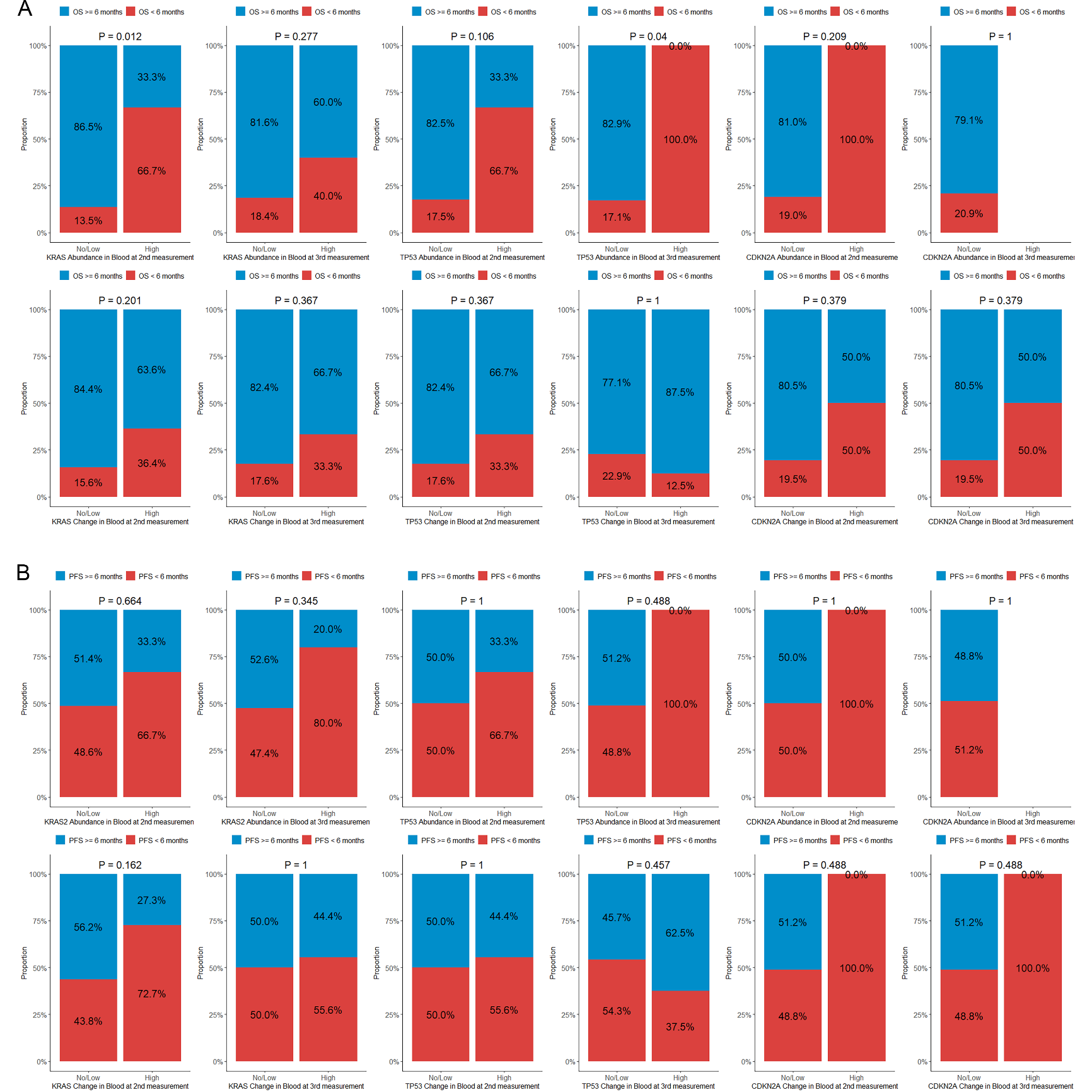


**Figure S4.** Proportions of cases with overall survival (OS; **A**) and progression-free survival (PFS; **B**) < versus ≥ 6 months in patients with high versus low/no mutations in ctDNA at repeated measurements.


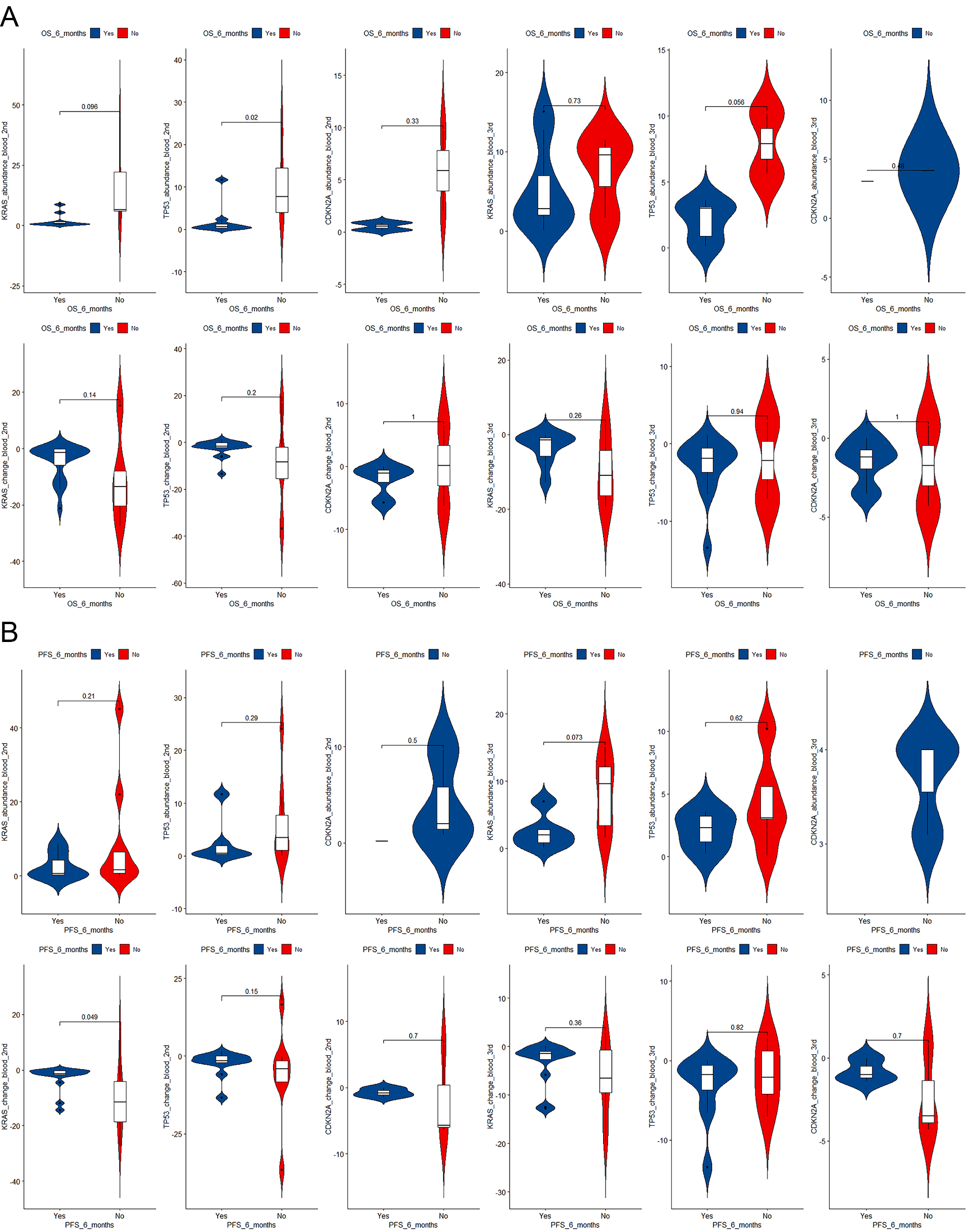


**Figure S5.** Differences in mutation abundances in ctDNA at repeated measurements between patients with overall survival (**A**) and progression-free survival (**B**) < versus ≥ 6 months.
